# Shared and Specific Patterns of Brain Functional Network Abnormalities in Patients With Idiopathic Dystonia and Across Subtypes

**DOI:** 10.1002/cns.70816

**Published:** 2026-04-07

**Authors:** Gang Liu, Jiana Zhang, Yiheng Gao, Shiyuan Gong, Yuhan Luo, Linchang Zhong, Zilin Ou, Zhicong Yan, Weixi Zhang, Kangqiang Peng, Huiming Liu, Qingmao Hu, Jinping Xu

**Affiliations:** ^1^ Department of Neurology, the First Affiliated Hospital Sun Yat‐Sen University, Guangdong Provincial Key Laboratory for Diagnosis and Treatment of Major Neurological Diseases, National Key Clinical Department and Key Discipline of Neurology Guangzhou China; ^2^ Institute of Biomedical and Health Engineering, Shenzhen Institutes of Advanced Technology Chinese Academy of Sciences Shenzhen China; ^3^ Department of Medical Imaging Sun Yat‐Sen University Cancer Center, State Key Laboratory of Oncology in Southern China, Collaborative Innovation Center for Cancer Medicine Guangzhou China; ^4^ Shenzhen University of Advanced Technology Shenzhen China

**Keywords:** functional networks, idiopathic dystonia, sensorimotor integration, subgroup analyses, topological analyses

## Abstract

**Aims:**

Accumulating neuroimaging evidence showed that idiopathic dystonia is a large‐scale network disorder, but whether the various clinical sub‐types shared imaging abnormalities remains controversial. We aimed to determine whether various forms of idiopathic dystonia have common topological changes or are distinct entities.

**Methods:**

Resting‐state functional magnetic resonance imaging and clinical data were obtained from 215 patients with various forms of dystonia and 160 healthy controls (HCs). Whole‐brain functional networks were constructed, and the topological parameters were calculated via graph theoretical analyses. Networks were compared between patients with idiopathic dystonia and HCs and in subgroup analyses (each dystonia subtype vs. HCs; one‐to‐one and one‐to‐many comparisons among blepharospasm [BSP], blepharospasm‐oromandibular dystonia [BOD], and cervical dystonia [CD] subgroups). Then, we analyzed the relationship between network topological changes and clinical characteristics in patients with idiopathic dystonia.

**Results:**

Compared to HCs, patients with idiopathic dystonia exhibited alterations in network integration and segregation. Subgroup analyses revealed similar changes in BSP and BOD but not in CD. Regionally, degree centrality and nodal efficiency (Ne) in the somatomotor network of patients with idiopathic dystonia decreased and increased in the subcortical and cerebellar networks. Decreased nodal clustering coefficient (Ncp) and nodal local efficiency were observed in the visual and subcortical networks. Similar regional alterations were observed in patients with BSP. Patients with idiopathic dystonia showed additional hub regions. Correlation analyses showed that higher Ne in visual and cerebellar networks correlated positively, and lower Ncp correlated negatively in the visual network with motor severity in patients with BSP.

**Conclusion:**

Our findings suggest that patients with BSP and BOD share extensive reorganization in the large‐scale functional network.

## Graphical abstract

The whole brain functional network based on brainnetome atlas with 28 cerebellar subregions was constructed and topological parameters were calculated and compared based on graph theoretical analyses between patients with idiopathic dystonia and healthy controls, as well as further subgroup analyses. Our findings suggest that patients with different forms of idiopathic dystonia might share extensive reorganization in the large‐scale functional network.

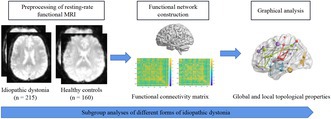

## 1 Introduction

Dystonia is a movement disorder characterized by sustained or intermittent muscle contractions that cause repetitive movements and/or abnormal postures in various body regions. Idiopathic adult‐onset dystonia, the most common form of dystonia, has variable clinical expressions; however, the underlying cause and pathophysiology of idiopathic dystonia remain incompletely understood.

The differences in epidemiological and clinical features reflect etiological disparities among the various clinical types of idiopathic dystonia. For example, blepharospasm (BSP) and cervical dystonia (CD) occur more frequently than laryngeal and limb dystonia [1]. Moreover, the age of onset varies among different forms of idiopathic dystonia. Dystonic symptoms appear earlier in the neck than in the face [2]. In addition, BSP experiences significantly more spread to adjacent body regions such as the oromandibular region than CD; however, sensory tricks are frequent in CD and less common in cranial dystonia [3]. Nevertheless, certain clinical features, such as spread, imply that the different forms of focal dystonia may be interrelated since various focal dystonias may coexist in the same individual due to spread [4]. In addition, neurophysiological studies revealed that shared abnormalities in the sensorimotor cortices were observed in various clinical types of dystonia [5, 6, 7, 8]. Currently, our pathophysiological understanding of idiopathic dystonia has been extensively improved using structural and functional magnetic resonance imaging (fMRI) and diffusion tensor imaging (DTI). Accumulating evidence from neuroimaging studies demonstrates the structural and functional changes in multiple brain regions, and the most common subtypes, such as focal hand, blepharospasm‐oromandibular dystonia (BOD), CD, and BSP may have shared imaging abnormalities, which included overactivity of the supplementary motor area and primary sensorimotor cortex disclosed by resting‐state fMRI (rs‐fMRI) and enlargement of basal ganglia and atrophy of thalamic nuclei [9, 10, 11, 12, 13, 14]. Recently, neuroimaging studies proposed that pathophysiological mechanisms underlying idiopathic dystonia can be better understood through a detailed knowledge of large‐scale network reorganization [15]. Our previous studies investigated the topological changes in anatomical networks based on DTI and graph theory analysis in BSP and CD patients. We found that although both patients with BSP and CD showed reorganization of large‐scale anatomical networks, the patterns of network reorganization of the two subtypes were completely inconsistent [16, 17]. However, whether various clinical types of idiopathic dystonia share connection changes in resting‐state functional networks, rather than those observed in specific anatomical structures, remains unknown.

In this study, we hypothesized that patients with BSP share topological alterations at the functional network level with those with BOD but not with patients with CD. To test our hypothesis, the whole‐brain functional network was constructed using the brainnetome atlas with 28 cerebellar subregions (BNA‐274 atlas). The topological properties of the functional networks were calculated and compared between patients with idiopathic dystonia and healthy controls (HCs), as well as between patients with BSP, BOD, CD, and HCs. Finally, we conducted a reproducibility analysis using an additional Anatomical Automatic Labeling atlas with 116 brain regions (AAL‐116), rSchaefer‐300, rSchaefer‐500, and rSchaefer‐700 atlases.

## 2 Methods

### 2.1 Participants

All patients were consecutively recruited from outpatient clinics for movement disorders between April 2019 and December 2024. Patients were included if they met the following criteria: (i) aged 18–75 years; (ii) diagnosed with idiopathic dystonia according to published diagnostic criteria by senior neurologists [18, 19, 20]. In contrast, the exclusion criteria were as follows: (i) botulinum toxin (BoNT) injection less than 3 months before MRI scans; (ii) oral medications less than 24 h before imaging; (iii) history of Alzheimer's disease, Parkinson's disease, traumatic brain injury, epilepsy, or stroke; (iv) history of antipsychotic‐induced tardive dyskinesia and hereditary dystonia and family history of movement disorders; (v) medical implants that were contraindicated for cerebral MRI. HCs matched with sex and age were recruited using the same exclusion criteria. Finally, 215 patients with idiopathic dystonia (102 with BSP, 43 with BOD, 56 with CD, four with craniocervical dystonia, four with writer's cramp, three with oromandibular dystonia, two with lower limb dystonia, and one with musician's dystonia) and 160 age‐ and sex‐matched HCs were recruited.

### 2.2 Clinical Assessment

Demographic and clinical characteristics, including sex, age, disease duration, and history of BoNT injections, were obtained through face‐to‐face interviews before MRI scanning. The motor severity of BSP, BOD, and CD were assessed using the Jankovic Rating Scale (JRS) [21], Burke‐Fahn‐Marsden Dystonia Rating Scale (BFMDRS) [22], and Toronto Western Spasmodic Torticollis Rating Scale (TWSTRS) [23], respectively. The nonmotor symptoms like anxiety and depression were evaluated using the Hamilton Anxiety Rating Scale (HAMA) [24] and Hamilton Depression Rating Scale (HAMD), respectively [25].

### 2.3 Image Acquisition

All resting‐state functional MRI (rs‐fMRI) data were acquired using a 3.0‐T Siemens scanner (Tim Trio; Siemens, Erlangen, Germany). Functional images were obtained using an echo‐planar imaging sequence with the following parameters: repetition time = 2000 ms, echo time = 30 ms, field of view = 220 mm × 220 mm, flip angle = 90°, voxel size = 3.4375 × 3.4375 × 4.6 mm^3^, 33 slices, and 240 volumes.

### 2.4 Imaging Preprocessing

Functional images were preprocessed using the Data Processing & Analysis for Brain Imaging toolbox (http:/rfmri.org/dpabi). The major steps included: (i) removing the initial 10 frames for data stability, followed by slice‐timing correction; (ii) aligning to the first volume to consider head motion, removing subjects with displacement over 3 mm or angular motion beyond 3 degrees; (iii) applying normalization for a resolution of 3 mm × 3 mm × 3 mm using the Montreal Neurological Institute (MNI) template; (iv) employing spatial smoothing through a 6 mm full‐width at half maximum (FWHM) Gaussian kernel; (v) conducting regression analysis on head motion parameters, average signals from cerebrospinal fluid and white matter; (vi) implementing temporal filtering within the 0.01–0.1 Hz band‐pass; and (vii) scrubbing with a preset criterion of frame displacement (FD) > 0.5.

### 2.5 Brain Network Construction

To construct a whole brain functional connectivity network (FCN) for each participant, the node was defined as Brainnetome atlas with 274 brain regions (BNA‐274, 246 cortical and subcortical regions plus 28 cerebellar regions; http://atlas.brainnetome.org/) [26]. Each region of cortices was labeled as the default model network (DMN), frontoparietal network (FPN), ventral attention network (VAN), dorsal attention network (DAN), limbic network (LN), somatomotor network (SMN), and visual network (VN), as well as the subcortical network and cerebellar network (Table S1) [27]. The functional connectivity defined by Pearson's correlation coefficient between the mean time series of each pair of the 274 regions was used to define the functional network edge. Finally, we obtained a symmetric 274 × 274 functional connectivity matrix for each participant for further analysis. Consistent with previous studies, we only used binary positive connectivity in subsequent analyses [28] based on the following considerations: (1) Binary networks prioritize “presence/absence” of connections over continuous strength, avoiding interference from spurious correlation fluctuations and allowing reliable capture of intrinsic global network properties (e.g., clustering coefficient, path length, modularity) [29, 30]. (2) Despite avoiding arbitrary thresholds, weighted networks are relatively susceptible to signal‐to‐noise fluctuations, and their thresholding must account for the impact of weak edges on topology [29]; such unstable weak connections may obscure target network architectures. Binary networks, by pruning spurious edges (e.g., via proportional thresholding ensuring consistent group density [31]), simplify patterns, focus on biologically meaningful strong connections, and directly reflect macroscale reorganization across dystonia subtypes and HCs. (3) Proportional thresholding in binary networks ensures uniform density across participants, eliminating confounding from group differences in overall connection strength [31]—critical for direct topological comparisons between subtypes and HCs without variable edge count interference. Weighted networks, while retaining more raw data, lack this consistency and may bias comparisons between groups with inherent connectivity magnitude differences. We acknowledge that binary networks sacrifice connection strength information [30, 31], but this is outweighed by our focus on investigating shared large‐scale network reorganization rather than quantifying intensity gradations.

### 2.6 Global Network Comparisons

Global network properties, including global efficiency (E_g_), local efficiency (E_loc_), clustering coefficient (C_p_), and characteristic path length (L_p_), were computed using the Gretna toolbox (https://helab.bnu.edu.cn/gretna/). E_g_ indicates the efficiency of information transmission across a network, whereas E_loc_ indicates how well a node exchanges information among the nodes [32]. L_p_ is the average shortest path length between any two nodes; a smaller L_p_ indicates faster information transfer to the entire brain region. C_p_ quantifies the prevalence of clustered connectivity around individual nodes, which is an important indicator for measuring the degree of network interconnection [33]. The differences in these global indexes between the idiopathic dystonia and HCs groups, with age, sex, and mean FD as covariates, were explored using two‐sample *t*‐tests. The significance level was set at *p* < 0.05 and corrected using the False Discovery Rate (FDR). The detailed methodologies and mathematical formulations of these global network metrics are proved in the Supporting Information.

### 2.7 Regional Network Comparisons

We calculated the degree centrality (DC), nodal efficiency (Ne), nodal clustering coefficient (Ncp), and nodal local efficiency (Nle) for all 274 regions to measure a node's role as a bridge along the shortest path between other nodes, reflecting its importance in controlling the information flow in the network. The DC provides insights into the number of direct connections of a node, indicating its potential influence on the network [34]. The Ne illustrates the efficiency of parallel information transfer for a given node in the brain network. The Nle, defined as the inverse of the shortest average path length in a subgraph comprising a node and its adjacent neighbors, is considered a measure of fault tolerance in a network because it characterizes how well information is exchanged by neighbors if the node is removed [35]. The Ncp measures the extent of interconnectivity among the neighbors of the node. Nle is similar to the NCp, reflecting the fault tolerance of networks [32]. The group differences in DC, Ne, Ncp, and Nle were explored and compared using two‐sample *t*‐tests between the two groups, with age, sex, and mean FD as covariates. The significance level was set at *p* < 0.05 and corrected using FDR. Additionally, a node was considered a hub region if its DC value was at least three standard deviation (SD) greater than the mean DC value for all nodes in a group (DC [*i*] > mean + 3SD).

### 2.8 Reproducibility Analyses

To explore the reproducibility of our findings, we employed an array of additional parcellation schemes to evaluate the potential effect of varying parcellation strategies on the whole brain using the AAL‐116, rSchaefer‐300, rSchaefer‐500, and rSchaefer‐700 atlases. Subsequently, the influence of differing threshold settings (0.05–0.50) on the network analysis results was scrutinized by adjusting threshold parameters [36].

### 2.9 Subgroup Analyses

Subgroup analyses of patients with idiopathic dystonia and HCs were conducted between patients with BSP, BOD, CD and the entire cohort of HCs (*n* = 160) to determine whether different forms of idiopathic dystonia shared topological alterations at the functional network level. In addition, to maintain more matching number and age distribution of the different subtypes of idiopathic dystonia, we adopted the subgroup analyses of global and local network properties between patients with BSP (*n* = 102) and age‐matched HCs (*n* = 102), BOD (*n* = 43) and age‐matched HCs (*n* = 43), as well as CD (*n* = 56) and age‐matched HCs (*n* = 56), respectively. Then, the comparison of global topological properties among the three subgroups of idiopathic dystonia (one‐to‐one and one‐to‐many) was further analyzed to explore the possible differences among the subtypes.

### 2.10 Statistical Analyses for Demographic and Clinical Characteristics

We analyzed the demographic and clinical characteristics of all participants using SPSS 25.0. Two sample *t*‐tests were used to identify differences in age and head movements between patients with idiopathic dystonia and HCs as well as in the subgroup analyses (BSP vs. HCs; BOD vs. HCs; CD vs. HCs). The χ^2^ test was used to compare sex differences, and Mann–Whitney *U* tests were used to verify differences in HAMA and HAMD scores between patients with idiopathic dystonia and across the subtypes and HCs, respectively. Additionally, the relationships between the theoretical metrics of abnormal network graph and disease duration, motor severity, HAMA, and HAMD scores were assessed using partial correlation analyses after adjusting for age, sex, and FD. Values of *p* < 0.05 were considered statistically significant.

## 3 Results

### 3.1 Demographic Information and Clinical Characteristics

In this study, 215 patients with idiopathic dystonia (137 females; mean age: 50 years) and 160 healthy controls (103 females; mean age: 48 years) were included. There were no significant differences in age, sex, or head motion between patients with idiopathic dystonia and HCs. Among the patients with idiopathic dystonia, the major subtypes of idiopathic dystonia belonged to the subgroups of craniocervical dystonia (102 with BSP, 43 with BOD, and 56 with CD). In the subgroup analyses between the idiopathic dystonia groups of the BSP, BOD, CD and the entire cohort of HCs (*n* = 160), there was a significant difference in age compared with the HCs (*p* < 0.001), whereas no significant differences in sex distribution or head motion were observed (Table 1).

**TABLE 1:** Subjects' demographics and clinical characteristics.

	Idiopathic dystonia (*n* = 215)	Subgroups of idiopathic dystonia			HCs (*n* = 160)	*P*	*P1*	*P2*	*P3*
		BSP (*n* = 102)	BOD (*n* = 43)	CD (*n* = 56)					
Sex (F/M)^a^	137/78	71/31	29/14	30/26	103/57	0.896	0.382	0.708	0.153
Age^b^	49.94 ± 12.37	53.69 ± 9.01	56.25 ± 10.94	40.33 ± 11.72	47.90 ± 13.27	0.127	< 0.001	< 0.001	< 0.001
FD^b^	0.16 ± 0.09	0.16 ± 0.09	0.16 ± 0.07	0.16 ± 0.07	0.16 ± 0.08	0.798	0.884	0.720	0.965
Disease duration	3.69 ± 4.07	3.29 ± 3.61	4.87 ± 4.36	3.07 ± 3.77	—	—	—	—	—
BoNT (Y/N)	132/83	44/58	22/21	15/41	—	—	—	—	—
Duration of BoNT	1.03 ± 2.06	2.83 ± 2.65	2.93 ± 2.59	1.77 ± 2.19	—	—	—	—	—
JRS total score	6.14 ± 1.43 (*n* = 145)	6.13 ± 1.21 (*n* = 102)	6.16 ± 1.88 (*n* = 43)	—	—	—	—	—	—
BFMDRS‐M score	—	—	7.32 ± 0.38 (*n* = 38)	—	—	—	—	—	—
TWSTRS total score	—	—	—	32.43 ± 10.60 (*n* = 49)	—	—	—	—	—
Median HAMA scores^c^	5.0 (0–23)	4.0 (0–22)	7.0 (0–21)	6.0 (0–23)	3.0 (0–20)	< 0.001	< 0.001	< 0.001	< 0.001
Median HAMD scores^c^	4 (0–24)	3.0 (0–24)	5.0 (0–19)	5.0 (0–19)	2.0 (0–13)	< 0.001	< 0.001	< 0.001	< 0.001

*Note:* Values for mean **±** standard deviations.

Abbreviations: BFMDRS‐M, Burke‐Fahn‐Marsden Dystonia Rating Scale movement scales; BoNT, botulinum toxins; FD, frame displacement; JRS, Jankovic Rating Scale; SD, standard deviation; TWSTRS, Toronto Western Spasmodic Torticollis Rating Scale.

^a^ *ᵡ* ^2^ test.

^b^ Two sample *t*‐tests.

^c^ Mann–Whitney *U* tests.

### 3.2 Between‐Group Differences in Global Network Properties

**FIGURE 1:**
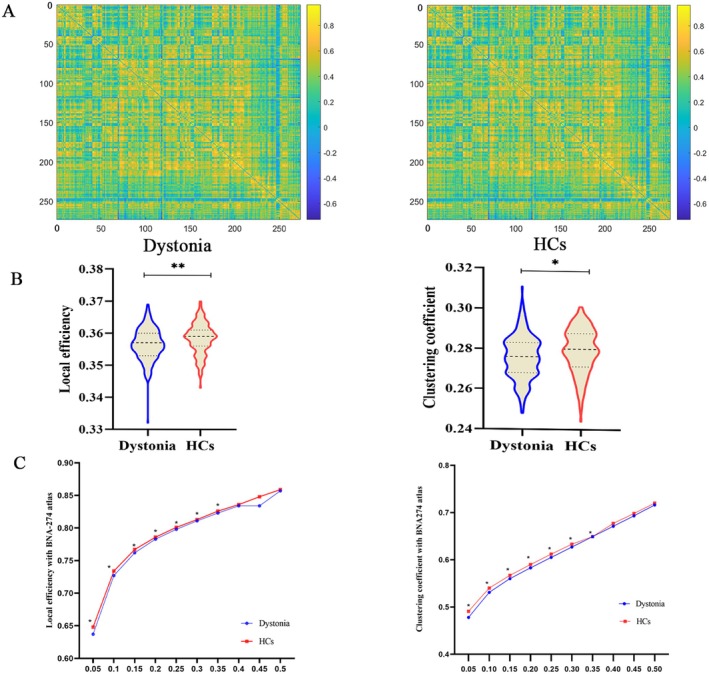
Comparison of global network properties between patients with idiopathic dystonia and HCs based on BNA‐274 atlas. (A) The symmetric 274 × 274 functional connectivity matrices of the idiopathic dystonia and HCs groups. (B) Comparison of local efficiency and clustering coefficient between idiopathic dystonia and HCs groups. (C) The comparison results of local efficiency and clustering coefficient as a function of sparsity (0.05–0.5) between patients with idiopathic dystonia and HCs. *FDR corrected *p* < 0.05. BNA‐274, Brainnetome atlas with 274 brain regions; FDR, False Discovery Rate; HCs, healthy controls.

We observed significantly decreased E_loc_ and C_p_ in patients with idiopathic dystonia compared with 160 HCs (Figure 1 and Table 2), with all metrics showing *p* < 0.05, corrected for FDR. In the subgroup analyses, significantly decreased E_loc_ and C_p_ and increased E_g_ were observed in patients with BSP, as well as decreased E_loc_, C_p_, and L_p_ and increased E_g_ in patients with BOD compared to HCs (Figure S1; Tables S2 and S3). However, no significant difference in the global indices was observed between patients with CD and HCs (Table S4).

**TABLE 2:** Comparison of global network properties between patients with idiopathic dystonia (*n* = 215) and HCs (*n* = 160).

	Dystonia (mean ± SD)	HCs (mean ± SD)	*T*	FDR corrected *p*
(a) BNA‐274				
E_loc_	0.356 ± 0.005	0.358 ± 0.005	−3.39	< 0.001^a^
E_g_	0.268 ± 0.005	0.267 ± 0.005	1.81	0.071
C_p_	0.276 ± 0.0109	0.279 ± 0.011	−2.79	0.006^a^
L_p_	0.793 ± 0.026	0.797 ± 0.026	−1.45	0.148
(b) AAL_116				
E_loc_	0.347 ± 0.008	0.348 ± 0.007	−1.97	0.060
E_g_	0.255 ± 0.009	0.254 ± 0.008	1.85	0.065
C_p_	0.277 ± 0.009	0.280 ± 0.010	−2.83	0.005^a^
L_p_	0.870 ± 0.051	0.878 ± 0.047	−1.71	0.089
(c) rSchaefer‐100				
E_loc_	0.348 ± 0.006	0.349 ± 0.006	−1.30	0.194
E_g_	0.260 ± 0.007	0.259 ± 0.007	1.44	0.150
C_p_	0.274 ± 0.009	0.276 ± 0.010	−6.16	< 0.001^a^
L_p_	0.841 ± 0.026	0.845 ± 0.037	−1.03	0.302
(d) rSchaefer‐300				
E_loc_	0.357 ± 0.001	0.359 ± 0.005	−2.80	0.005^a^
E_g_	0.269 ± 0.005	0.268 ± 0.005	1.85	0.060
C_p_	0.276 ± 0.001	0.279 ± 0.011	−2.61	0.009^a^
L_p_	0.789 ± 0.024	0.7926 ± 0.023	−1.67	0.096
(e) rSchaefer‐500				
E_loc_	0.358 ± 0.001	0.360 ± 0.005	−3.43	< 0.001^a^
E_g_	0.272 ± 0.001	0.271 ± 0.004	2.17	0.031^a^
C_p_	0.275 ± 0.001	0.279 ± 0.012	−2.97	0.003^a^
L_p_	0.774 ± 0.001	0.777 ± 0.018	−1.99	0.047^a^
(f) rSchaefer‐700				
E_loc_	0.359 ± 0.001	0.362 ± 0.006	−3.51	< 0.001^a^
E_g_	0.273 ± 0.001	0.272 ± 0.004	2.16	0.032^a^
C_p_	0.275 ± 0.001	0.279 ± 0.013	−3.09	0.002^a^
L_p_	0.768 ± 0.017	0.771 ± 0.0164	−1.88	0.061

Abbreviations: C_p_, clustering coefficient; E_g_, global efficiency; E_loc_, local efficiency; HCs, healthy controls; L_p_, characteristic path length.

^a^ *p* < 0.05.

### 3.3 Between‐Group Differences in Local Network Properties

**FIGURE 2:**
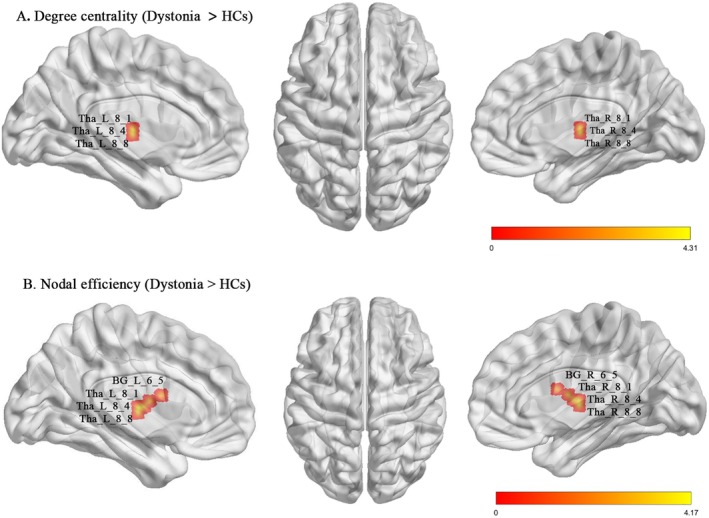
Increased local topological changes in the regions of cortical and subcortical networks between patients with idiopathic dystonia and HCs. *FDR corrected *p* < 0.05. FDR, False Discovery Rate; HCs, healthy controls.

**FIGURE 3:**
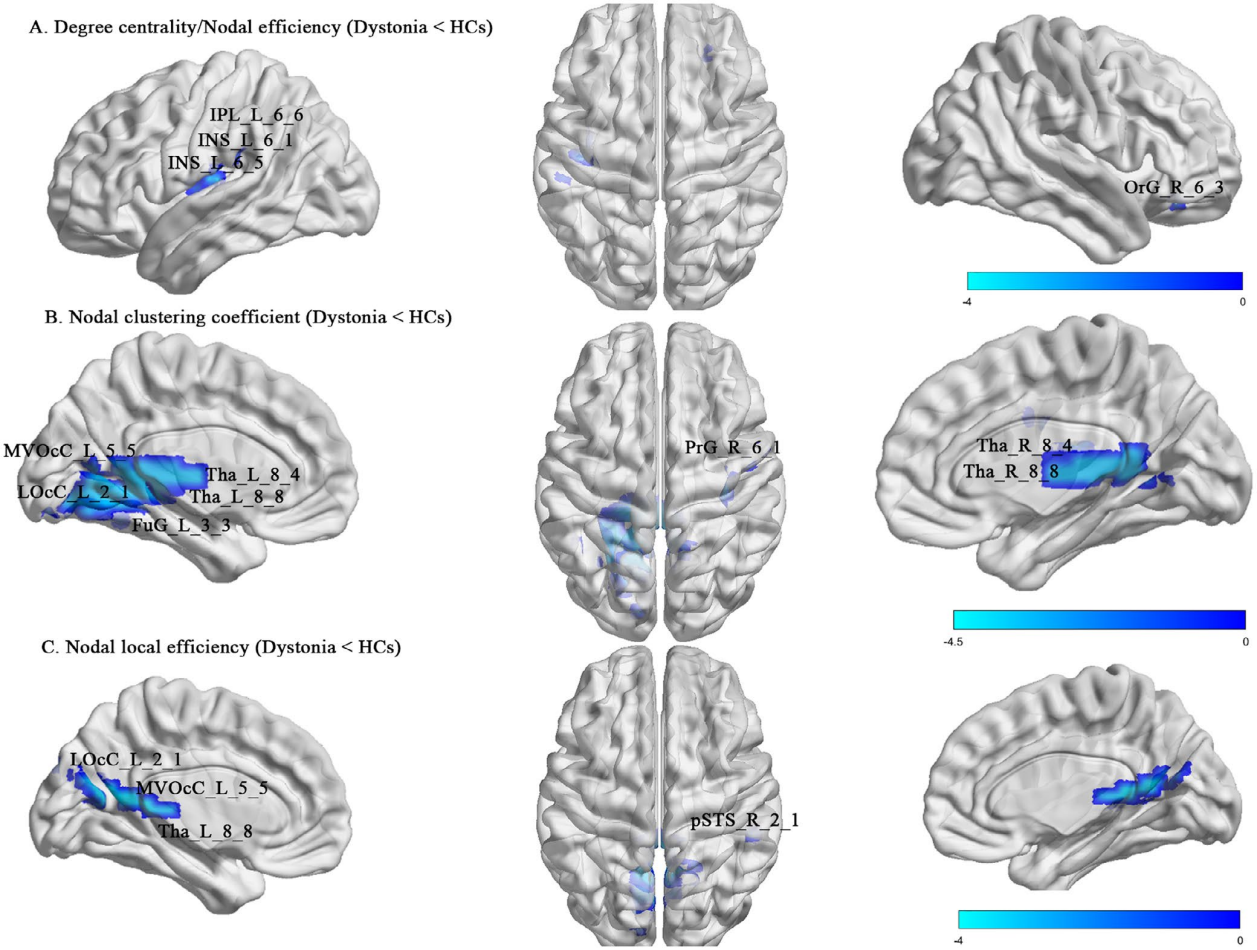
Decreased local topological changes in the regions of cortical and subcortical networks between patients with idiopathic dystonia and HCs. *FDR corrected *p* < 0.05. FDR, False Discovery Rate; HCs, healthy controls.

Compared with the entire cohort of HCs (*n* = 160), patients with idiopathic dystonia had significantly decreased DC and Ne values in several regions, mainly located in the somatomotor network, and increased DC and Ne values in the subcortical and cerebellar networks. Also, decreased Ncp and Nle values were observed in the regions of the visual and subcortical networks (*p* < 0.05, FDR corrected; Figures 2 and 3 and Table S5). Similarly, patients with BSP showed significantly higher DC and Ne values in the main regions of the subcortical and cerebellar networks, with lower Ncp and Nle values in the visual and subcortical networks than HCs (*p* < 0.05, FDR corrected; Table S6). Additionally, patients with CD showed increased DC in the subcortical and cerebellar networks (*p* < 0.05, FDR corrected; Table S7).

### 3.4 Network Hub Distributions

**FIGURE 4:**
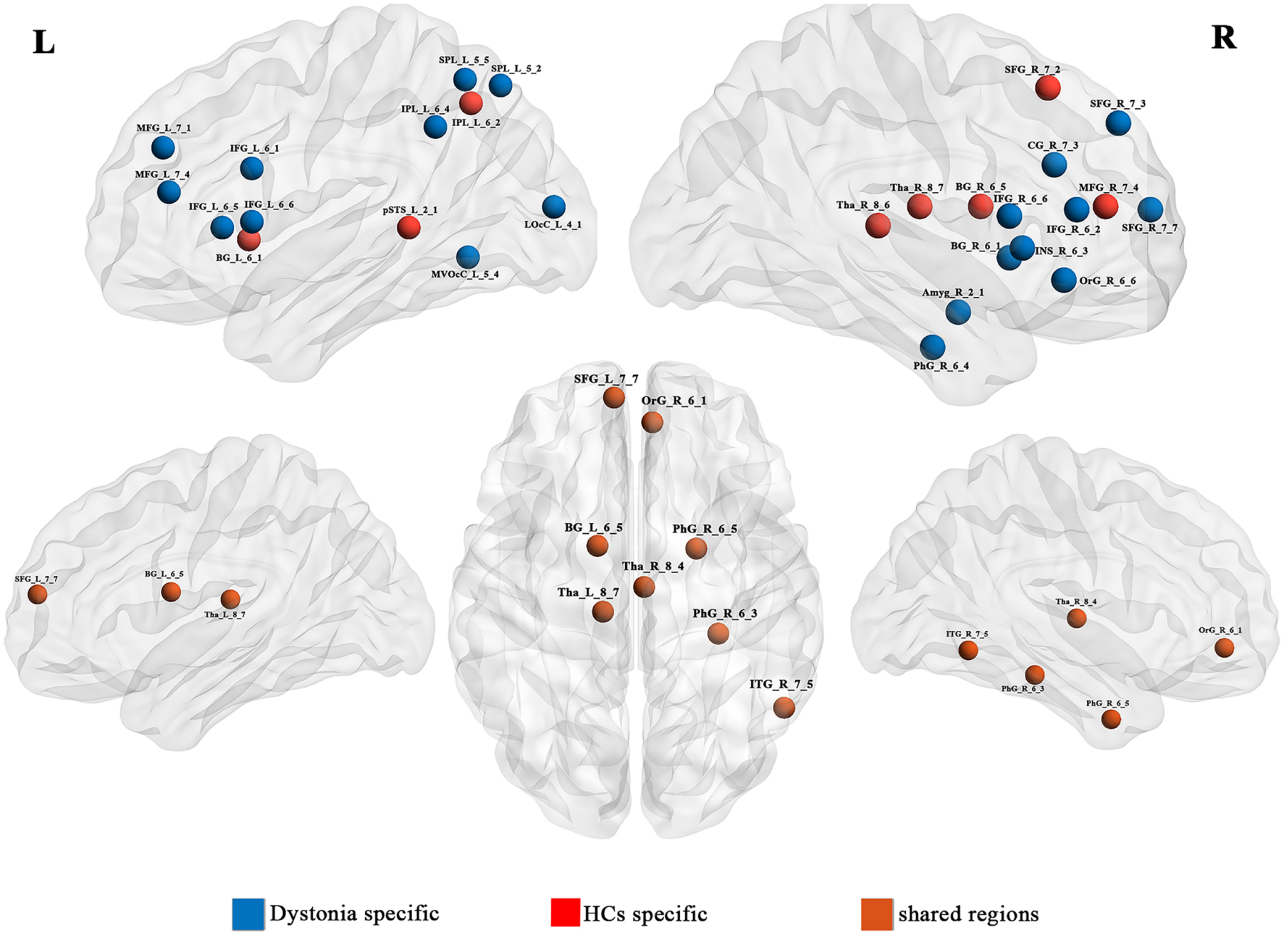
Distributions of hub regions based on DC in patients with idiopathic dystonia and HCs. Specific and shared hubs in idiopathic dystonia and HCs are marked using blue, red, and blown, respectively. DC, degree centrality; HCs, healthy controls.

Details of the hub regions of the functional network in patients with idiopathic dystonia and HCs are presented in Figure 4 and Table S8. There were 34 and 19 hub regions in the idiopathic dystonia and HCs groups, respectively. Among these, nine regions were shared hubs between the two groups. Moreover, hub distributions in the two groups were similar to those in the main regions located in the frontoparietal, default, subcortical, and cerebellar networks. In the subgroup analyses, similar specific hub regions were observed in patients with BSP (Table S9), but not in patients with BOD and CD.

### 3.5 Between‐Group Differences as Compared to Age‐Matched HCs, Respectively

Further analyses revealed no significant differences in age, sex, or head motion between three subtypes of idiopathic dystonia and age‐matched HCs groups (Table S10).

Furthermore, significantly decreased E_loc_ and C_p_ and increased E_g_ were observed in patients with BSP, and increased E_g_ in patients with BOD compared to age‐matched HCs (*p* < 0.05, corrected for FDR; Figure S2 and Tables S11 and S12). Meanwhile, no significant difference in the global properties was observed between patients with CD and age‐matched HCs (Table S13).

Moreover, patients with BSP showed significantly increased DC and Ne values in the cerebellar network, and decreased DC and Ne values in the somatomotor network (*p* < 0.05, corrected for FDR; Table S14) compared to their age‐matched HCs (*n* = 102); whereas there was no significant alteration in patients with BOD and CD compared with their age‐matched HCs, respectively.

### 3.6 Comparison of Global Topological Properties Among the Three Subgroups of Idiopathic Dystonia

Comparison of global topological properties among the three dystonia subgroups revealed no significant differences (Table S15, *p* > 0.05), including one‐to‐one direct comparisons (BSP vs. BOD, BSP vs. CD, and BOD vs. CD) and one‐to‐many direct comparisons (BSP vs. BOD&CD, BOD vs. BSP &CD, and CD vs. BSP &BOD).

### 3.7 Correlations Analyses

No significant correlations between altered topological properties and disease duration were observed in patients with idiopathic dystonia or the BSP, BOD, and CD subgroups. As for the motor severity in patients with BSP, increased Ne in the left ventromedial parietooccipital sulcus (MVOcC_L_5_5; *n* = 102, *r* = 0.203, *p* = 0.044) and Cerebellum_V_IX (*n* = 102, *r* = 0.239, *p* = 0.017) were found to be positively correlated with JRS total score. In contrast, decreased Ncp in the MVOcC_L_5_5 (*n* = 102, *r* = −0.308, *p* = 0.002) was negatively correlated with JRS total score (Table S16 and Figure S3). No significant correlations were observed between altered topological properties and either motor severity or nonmotor symptoms in the BOD and CD subgroups (Tables S17 and S18).

### 3.8 Reproducibility of Results

We repeated the reconstruction of the functional network using the AAL‐116, rSchaefer‐100, rSchaefer‐300, rSchaefer‐500, and rSchaefer‐700 atlases. Compared to HCs, patients with idiopathic dystonia showed similarly decreased C_p_ in the AAL‐116 and rSchaefer atlases, low E_loc_ and C_p_ in the rSchaefer‐300, rSchaefer‐500, and rSchaefer‐700 atlases, and high E_g_ in the rSchaefer‐500 and rSchaefer‐700 atlases (Table 2, Tables S2–S4, and Tables S11–13).

## 4 Discussion

This study investigated the complex functional network organization based on global and regional alterations in patients with idiopathic dystonia using a graphical analysis. Compared with HCs, patients with idiopathic dystonia showed disrupted segregation and more integration and interconnection of the subcortical and cerebellar networks, which primarily displayed abnormal sensorimotor integration by altered DC and regional efficiencies (Ne and Nle) of the SMN, subcortical, and cerebellar networks. Our findings demonstrate that idiopathic dystonia is a large‐scale network disorder involving extensive cortical, subcortical, and cerebellar regions located in basal ganglia‐ cerebellar‐thalamo‐cortical circuits. Moreover, the BSP and BOD subgroups showed similar network organization results, consistent with the hypothesis that there might be shared mechanisms between the different subtypes of dystonia [4].

In the global‐level analyses, patients with idiopathic dystonia showed significantly decreased E_loc_ and C_p_, indicating impaired segregation of the whole‐brain functional network [32]. E_loc_ is closely related to C_p_, regarded as the index of segregation and reflecting the average efficiency of local clusters in the structural and functional networks. Higher E_loc_ and C_p_ indicate densely locally connected clusters [37]. Therefore, lower E_loc_ and C_p_ may jointly indicate decreased network segregation in idiopathic dystonia. Moreover, in the subgroup analyses, disrupted segregation was found in patients with BSP and BOD, suggesting a common mechanism for different forms of idiopathic dystonia. Previous studies have shown consistent neural activity abnormalities and microstructural alterations of the sensorimotor cortex, basal ganglia, thalamus, and cerebellum among different subtypes of focal dystonia [38, 39]. Moreover, we observed increased E_glob_ in patients with BSP, as well as higher E_glob_ and lower L_p_ in patients with BOD, revealing a more efficient transmission and hyperactive functional integration, which is consistent with the alterations of the white matter network in patients with BSP, as shown in our previous study [17]. Although no significant global alterations were observed in patients with CD, increased E_g_ and decreased E_loc_ and C_p_ were observed, indicating the more integration and disassortative network, contrary to our previous findings regarding microstructural network in patients with CD [16]. This phenomenon may be associated with the concrete map partitioning of the atlases and analytical methodology. In our previous structural network topological analyses in patients with CD, we used the BNA‐246 atlas, which lacks infratentorial structures such as the cerebellum and brainstem. However, accumulating evidence confirms that the cerebellum plays a pivotal role in CD pathogenesis [40]. To address this limitation, the current study adopted the BNA‐274 atlas including 28 cerebellar regions, with the results reflecting the possible compensatory functional adjustments driven by cerebellar network abnormalities. Moreover, differences in the network organization of structural connectivity and functional connectivity were reported. Structural networks tend to form assortative architectures, where nodes with similar connection strengths (e.g., high‐degree hubs) are preferentially linked. In contrast, functional networks adopt a disassortative organization, where high‐strength nodes are relatively likely to connect with low‐strength nodes [41]. This configuration restricts the indiscriminate propagation of abnormal neural activity and maintains functional stability by preventing cascading disruptions. Therefore, in our network changes in patients with CD, the structural network exhibits increased assortativity [16], while the functional network shows more integration and disassortativity. This contrast aligns with the inherent division of labor between structural connectivity and functional connectivity, reflecting the brain adaptive balance between structural stability and functional flexibility [41].

From the perspective of the regional‐level analysis, we identified significantly decreased DC and Ne values in SMN, whereas increased DC and Ne values were observed in the subcortical and cerebellar networks. The SMN involves sensory processing and action execution, which are observed with microstructural abnormalities and hypoconnectivity in patients with BSP and CD [39, 42], indicating sensorimotor integration dysfunction in focal dystonia. Furthermore, within the sensorimotor integration loop, subcortical regions, such as the basal ganglia, thalamus, and cerebellum, initially encode the sensory inflow, with processed signals subsequently relayed to the somatosensory cortex, which in turn projects to the primary motor cortex, thereby calibrating the motor output [43]. Ni et al. found that increased functional connectivity between the right striatum and the supplementary motor cortex might result in sensory errors from the basal ganglia and then cause erroneous motor output in patients with BSP [44]. Therefore, in our analysis, the increased DC and Ne in subcortical regions, such as the thalamus and cerebellum in patients with idiopathic dystonia (associated with enhanced local information transmission efficiency), suggest a sensory input error and hyperconnectivity in the thalamus and cerebellum regions, which may lead to abnormal motor information transfer, as reflected by decreased DC and Ne in the sensorimotor cortex. This finding is consistent with previous studies that demonstrated that the inhibitory function of the motor cortex from the cerebellum is reduced in focal dystonia [45, 46]. Further, the decreased Nle and Ncp jointly indicate reduced segregation and weaker connectivity with neighboring regions, primarily observed in the thalamus and visual cortices, especially in the medial ventral occipital cortex (MVOcC) and lateral occipital cortex (LOcC) regions; this suggests, a compensatory mechanism of functional integration in the visual network and thalamus [46]. Subgroup analyses revealed similar local alterations based on the increased DC and Ne in subcortical and cerebellar network regions, together with decreased Nle and Ncp in the thalamus and visual cortices in patients with BSP. Increased DC was observed in some partial regions in patients with CD, suggesting that subtypes of idiopathic dystonia may share common patterns of functional connectivity changes.

The correlation analyses reveal that increased Ne in the MVOcC_L_5_5 and Cerebellum_V_IX, along with decreased Ncp in the MVOcC_L_5_5, correlate with worse motor severity in patients with BSP. These findings suggested that the enhanced global information integration (via increased Ne) in visual and cerebellar networks likely represents a compensatory adaptation to motor dysfunction in patients with BSP, while reduced local connectivity (lower Ncp) in the ventromedial parietooccipital sulcus indicates impaired regional signal processing critical for fine motor control [45]. Wang et al. [47] demonstrated that altered nodal efficiency in motor cortex correlates with motor severity in craniocervical dystonia, and our analyses extend this observation by highlighting the regions in visual and cerebellar networks, which belong to the key visuomotor integration hub and engage in motor coordination [45, 48]. Consistent with previous research showing sensorimotor cortex abnormalities in BSP, our finding of reduced local clustering in the MVOcC_L_5_5 underscores disrupted regional processing in circuits governing eyelid movement [49].

In addition, our study demonstrated that patients with idiopathic dystonia exhibited additional hub regions in the whole brain, primarily concentrated in the cortical networks (VN, DAN, VAN, FPN, and DN), basal ganglia, thalamus, and cerebellum. Hubs are regions with high connectivity located in the most efficient communication pathways. The distribution of hub regions in patients with idiopathic dystonia suggests a dystonia‐specific reorganization of functional networks and potential pathophysiology related to cerebellar‐basal ganglia‐thalamus‐cortical circuits [45]. It has been reported that cerebellar dysfunction plays a vital role in motor behavior in animal models of dystonia, potentially driven by defects at the climbing fiber‐Purkinje cell synapse [50]. The thalamus is an important integrity region that integrates input information from the cerebellum and basal ganglia, which are represented as intermediate structures to the cortex [51]. It was observed that decreased structural connectivity of thalamic, occipital, and motor cortices in patients with BSP supports the pathology of abnormal visual sensory processing and motor dysfunction, further demonstrating that basal ganglia and thalamus abnormalities can be induced by aberrant cerebellar afferent projections [52, 53]. Eye symptoms such as photophobia are frequently observed in patients with BSP, and visual network abnormalities were thought to be potentially associated with the symptom of photophobia, particularly in relation to specific premonitory or accompanying symptoms in migraine [54]. Recent studies have provided evidence for dynamic alterations in spontaneous cortical neural activity and increased activation of the primary visual cortex underlying light sensitivity in chronic migraine under light stimulation. These findings have facilitated the objective assessment of photophobia and associated neural activity changes in the visual network [55, 56]. However, in our current study, alterations in visual network hubs in patients with BSP were analyzed using rs‐fMRI, rather than task‐based fMRI with synchronous visual stimulation. This methodological difference means our findings may not specifically reflect visual network hubs associated with photophobia in patients with BSP.

Reproducibility analyses showed that patients with idiopathic dystonia demonstrated significantly decreased E_loc_ and C_p_ in the rSchaefer‐300 atlas, and increased E_g_ in rSchaefer‐500 and rSchaefer‐700 atlases. Furthermore, similar integrated and segregated alterations in functional networks were observed in the BSP and BOD subgroups. Additionally, local topological alterations were observed based on DC and Ne in patients with BSP, implying the robustness of these findings. The rSchaefer atlases with multiresolution parcellations are functionally and connectionally homogeneous, providing reference atlases for graph theoretic analysis and neural mass modeling [57].

This study had certain limitations. First, most patients with idiopathic dystonia in our study had BSP (102 out of 215 patients), implying that our findings may be more closely associated with the pathophysiological mechanisms of BSP. Second, the effect of BoNT injections and oral medications on functional alterations is currently unclear and should be considered in future studies. Third, photophobia and other visual disturbances are common nonmotor symptoms of BSP and visual network abnormalities were thought to be potentially associated with the symptom of photophobia [58, 59]. However, we did not perform the clinical evaluation scales of photophobia or other eye symptoms [58, 60]. Consequently, we cannot currently provide evidence supporting a specific association between visual network hub alterations and visual disturbances in patients with BSP. Fourth, no significant correlations were observed between functional alterations and disease duration, motor severity, and nonmotor symptoms (e.g., anxiety and depression) in patients with BOD and CD, which may be attributed to relatively small sample sizes and incomplete motor assessment data in these cohorts.

In conclusion, by comparing the topological features between patients with idiopathic dystonia and HCs, we identified regional and global alterations in the functional networks of patients with idiopathic dystonia. Our findings indicate that idiopathic dystonia exhibits extensive brain reorganization at the whole‐brain network level. A detailed understanding of this large‐scale network reorganization could assist researchers in better comprehending the neuro‐mechanisms of idiopathic dystonia and foster the development of therapeutic strategies. Specifically, the therapeutic modulation of network regions (such as the visual network, thalamus, and cerebellum) through noninvasive or invasive stimulation techniques could potentially improve idiopathic dystonia symptoms.

## Author Contributions

Gang Liu: data curation, conceptualization, methodology, writing – original draft, writing – review and editing, and funding acquisition. Jiana Zhang: methodology, formal analysis, writing – original draft, writing‐review and editing. Yiheng Gao: methodology, formal analysis, writing – original draft, writing – review and editing. Shiyuan Gong: data curation, writing – review and editing, and software. Yuhan Luo: data curation, writing – review and editing, and software. Linchang Zhong: data curation, methodology, software, and visualization. Zilin Ou: writing – review and editing and data curation. Zhicong Yan: writing – review and editing and data curation. Weixi Zhang: conceptualization, resources, writing‐review and editing, and data curation. Kangqiang Peng: data curation, methodology, and software. Huiming Liu: data curation, methodology, software, and visualization. Qingmao Hu: conceptualization, resources, data curation, and investigation. Jinping Xu: conceptualization, resources, writing‐review and editing, funding acquisition, supervision, data curation, and investigation.

## Funding

This work was funded by the National Natural Science Foundation of China (62476267, 81771137, 81971103, 82101399, 82271300, and 82471258), Natural Science Foundation of Guangdong Province (2024A1515030183, 2023A1515012739 and 2021A1515010600), Guangdong Key Project (2018B030335001), Guangzhou Key Project (202007030002), Guangzhou Science and Technology Plan (Industry‐University‐Research Cooperation) (2023B03J0466), Guangdong Provincial Clinical Research Center for Neurological Diseases (2020B1111170002), Guangdong Province International Cooperation Base for Early Intervention and Functional Rehabilitation of Neurological Diseases (2020A0505020004), Guangzhou Major Difficult and Rare Diseases Project (2024MDRD02), Shenzhen Medical Research Fund (A2503031), Guangdong Provincial Engineering Center for Major Neurological Disease Treatment, and Guangdong Provincial Translational Medicine Innovation Platform for Diagnosis and Treatment of Major Neurological Disease.

## Ethics Statement

We declare that the formal approval to conduct the experiments described was obtained by the Ethical Committee of the First Affiliated Hospital of Sun Yat‐Sen University [(2020)323] and can be provided upon request.

## Conflicts of Interest

The authors declare no conflicts of interest.

## Supporting information


**Data S1:** cns70816‐sup‐0001‐Supinfo.docx.

## Data Availability

The data that support the findings of this study are available from the corresponding author upon reasonable request.
