# Supplementary material for: Shared and Specific Patterns of Brain Functional Network Abnormalities in Patients With Idiopathic Dystonia and Across Subtypes
Source: CNS Neurosci Ther. 2026 Apr 7;32(4):e70816. doi: 10.1002/cns.70816 (PMC13056703; doi:10.1002/cns.70816)
Supplement: Supplementary file 1 — Data S1: cns70816‐sup‐0001‐Supinfo.docx. [file CNS-32-e70816-s001.docx]

**SUPPLEMENTAL MATERIAL**

**Network analysis**

To delineate the topological architecture of the functional connectivity network (FCN), we employed the Gretna toolbox ([https://helab.bnu.edu.cn/gretna/](https://helab.bnu.edu.cn/gretna/)) to compute global efficiency (E_g_), local efficiency (E_loc_), clustering coefficient (C_p_), and the path length (L_p_), in addition to betweenness centrality (BC) and degree centrality (DC).

Suppose there are *N* regions with a certain communication range in the FCN. We define *G* = (*N, E*) as the communication topology of FCN, where *N* is the set of nodes and *E* is the set of edges in the network.

**1) Global efficiency:**

The concept of global efficiency^1^, serves as a measure of the network's capacity for parallel information transmission, which is defined in the following manner:

$$E_{\text{g }}(G)=\frac{1}{N(N-1)}\sum_{i\neq j\in G} \frac{1}{d_{ij}}$$

where *d_ij_*​ -represents the minimum path length between nodes *i* and *j* within graph *G*.

**2) Local efficiency:**

The concept of local efficiency^1^ is indicative of the network's fault tolerance, reflecting the efficiency of information exchange within each subgraph in the absence of the index node, which is defined in the following manner:

$$E_{\text{loc }}(G)=\frac{1}{N}\sum_{i\in G} E_{g}\left(G_{i}\right)$$

where *Eg*(*G_i_*) represents the global efficiency of *G_i_*, the subgraph comprising the neighbors of node *i*.

**3) Clustering coefficient:**

The clustering coefficient^1^ for a given node *i* is determined by dividing the total number of actual links by the total possible links that could exist among the neighbors of that node:

$$C_{i}=\frac{2E_{i}}{K_{i}\left(K_{i}-1\right)}$$

where *K_i_* represents the count of links connected to node *i*, and *E_i_* signifies the quantity of links present among its neighbors.

The network's clustering coefficient is computed by averaging the clustering coefficients across all nodes, which quantifies the degree of local clustering or the efficiency of local information exchange within the network. The clustering coefficient is defined in the following manner:

$$C_{p}=\frac{1}{N}\sum_{i\in G} C_{i}$$

**4) The path length:**

The definition of the shortest path length for a node within the network G (N, E) is defined in the following manner:

$$L_{i}=\frac{1}{N-1}\sum_{i\neq j\in G} d_{ij}$$

L_p_ represents the mean shortest path length amongest the nodes, which is defined in the following manner^1^:

$$L_{p}=\frac{1}{N}\sum_{i\in G} L_{i}$$

**5) Betweenness centrality**

The betweenness centrality^2^ of a node quantifies its role in facilitating information transfer across the network by connecting other node pairs, which is defined in the following manner:

$$BC(i)=\sum_{m\neq i\neq n\in G} \frac{S_{mn}(i)}{S_{mn}}$$

where *S_mn_* denotes the aggregate of shortest paths between nodes *m* and *n*, and *S_mn_*(*i*) represents the count of those paths traversing through node *i*.

**6) Degree centrality**

Degree centrality^3^ is a fundamental measure in network analysis that reflects the importance or influence of a node within the network. A node with high degree centrality acts as a critical hub, indicating it has numerous connections and potentially plays a significant role in the network's connectivity and communication, which is defined in the following manner:

$$DC(i)=\deg(i)$$

where *deg*(*i*) typically represents the degree of node *i*, which is the number of edges directly connected to node *i*.

For a standardized metric and a means of comparison across all network groups^4^, we utilized the normalized integrals of the network parameters:

$$\frac{\int_{a}^{b} E_{\text{g }}}{b-a},\frac{\int_{a}^{b} E_{\text{loc }}}{b-a},\frac{\int_{a}^{b} C_{p}}{b-a},\frac{\int_{a}^{b} L_{p}}{b-a},\frac{\int_{a}^{b} BC(i)}{b-a},\frac{\int_{a}^{b} DC(i)}{b-a}$$

where a and b are respectively the lower and upper limits of sparsity (a = 0.05 and b = 0.5).

**Supplemental references**

1. Achard S, Bullmore E. Efficiency and cost of economical brain functional networks. PLoS computational biology. 2007;3:e17.

2. Freeman L C. A set of measures of centrality based on betweenness. Sociometry. 1977;35-41.

3. Borgatti S P, Everett M G. A graph-theoretic perspective on centrality. Social networks. 2006;28:466-484.

4. Xu J, Zhang J, Zhang J, et al. Abnormalities in structural covariance of cortical gyrification in Parkinson's disease. Frontiers in Neuroanatomy. 2017;11:12.

**Supplemental figures**

**Figure S1. Comparison of global network properties between patients with BSP (n = 102) and the entire cohort of 160 HCs (A), patients with BOD (n = 43) and HCs (n =160, B) based on BNA-274 atlas, respectively.** *FDR corrected *P* < 0.05. Abbreviations: BNA-274, Brainnetome atlas with 274 brain regions; BOD, blepharospasm-oromandibular dystonia; BSP, blepharospasm; FDR, False Discovery Rate; and HCs, healthy controls. **P* < 0.05.

**
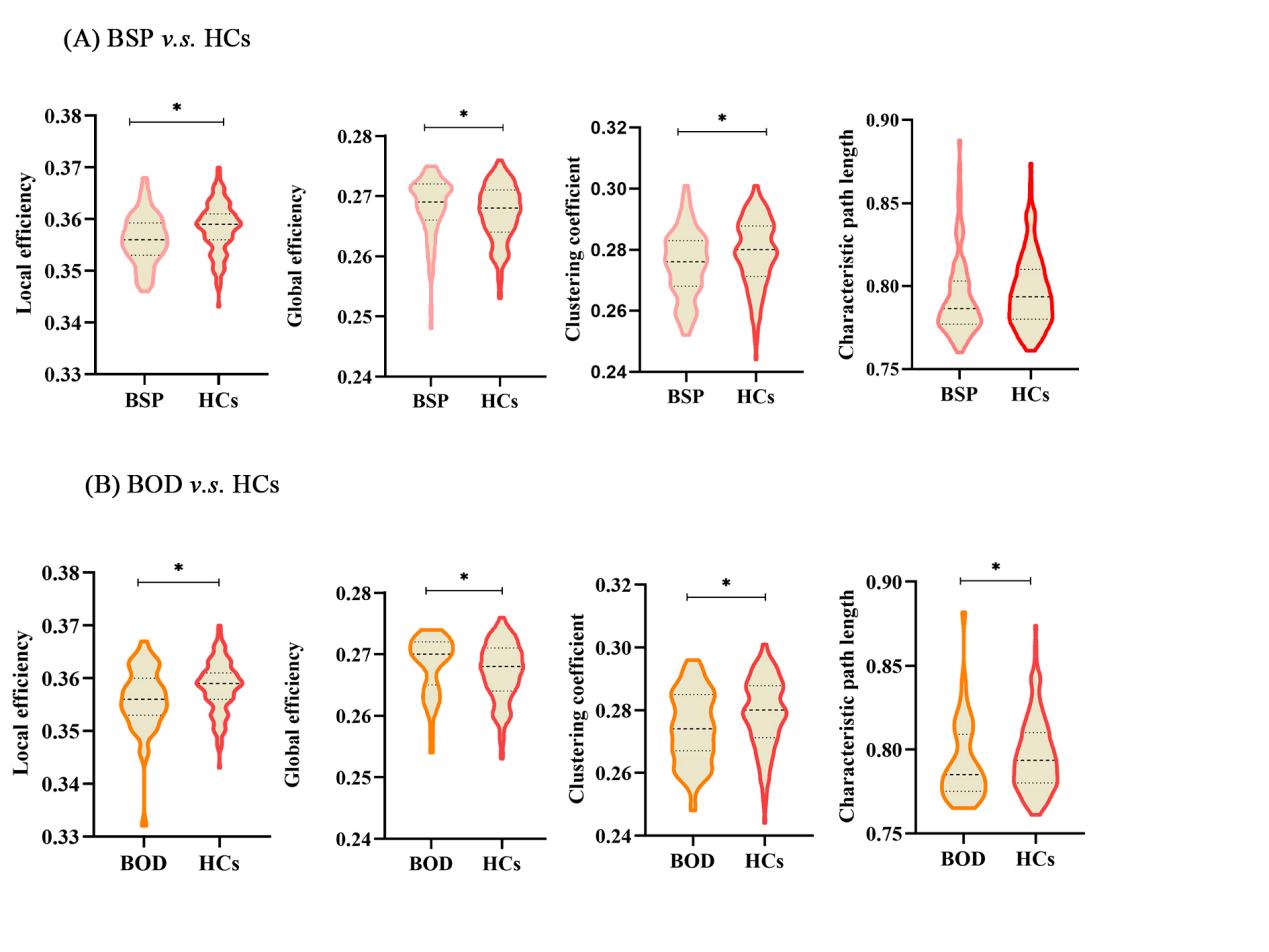
**

**Figure S2. Comparison of global network properties between patients with BSP (n = 102) and age-matched HCs (n = 102, A), as well as patients with BOD and age-matched HCs (n = 43, B) based on BNA-274 atlas, respectively.** *FDR corrected *P* < 0.05. Abbreviations: BNA-274, Brainnetome atlas with 274 brain regions; BOD, blepharospasm-oromandibular dystonia; BSP, blepharospasm; FDR, False Discovery Rate; and HCs, healthy controls. **P* < 0.05.

**
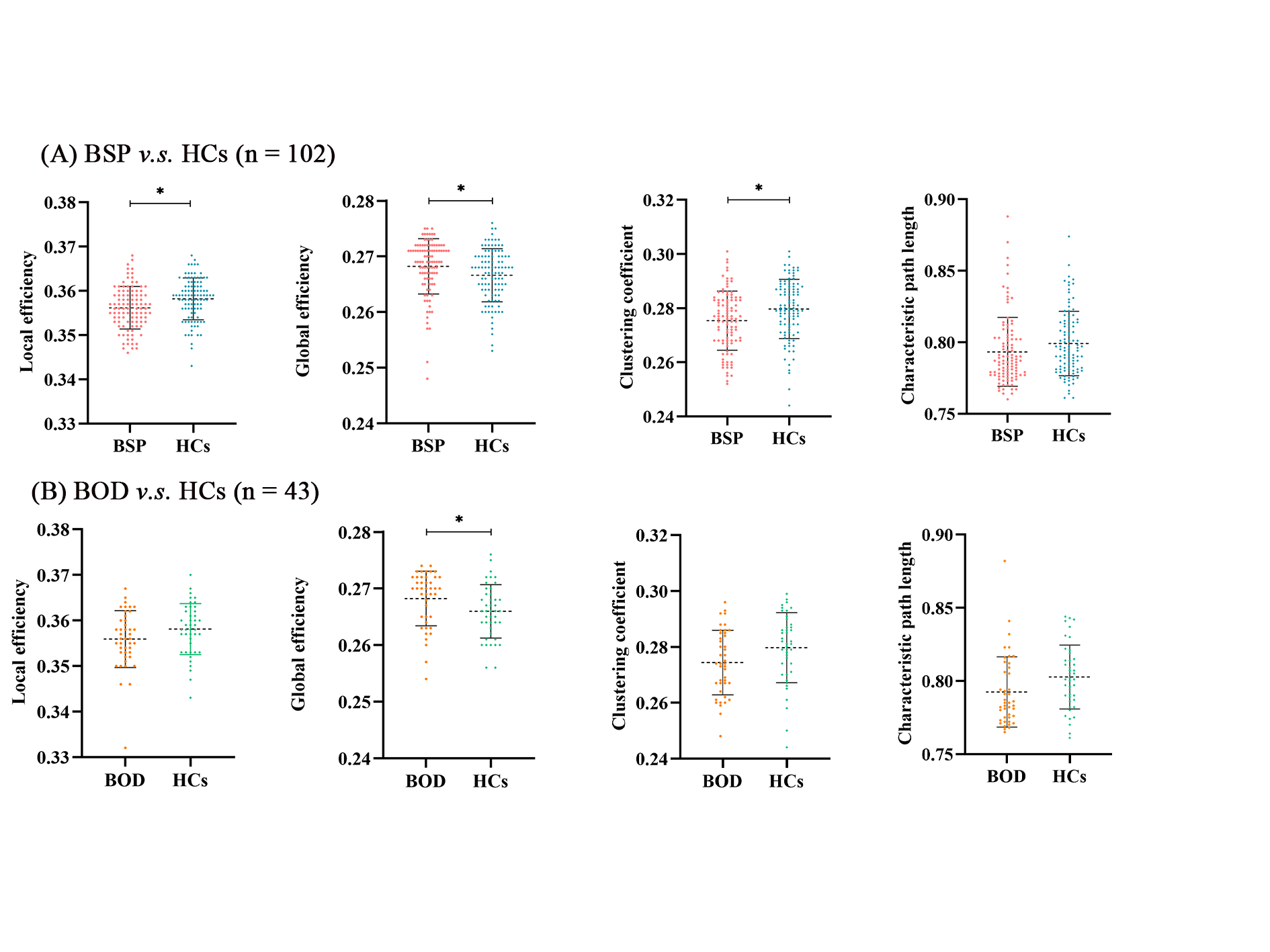
**

**Figure S3. Correlation results.** In the BSP subgroup, it was showed showed that increased nodal efficiency in the MVOcC_L_5_5 (n = 102, r = 0.203, *P* = 0.044) and right Cerebellum_V_IX (n = 102, r = 0.239, *P* = 0.017) was positively correlated with JRS total score (A, B). In contrast, decreased nodal clustering coefficient in the left MVOcC_L_5_5 (n = 102, r = -0.308, *P* = 0.002) was negatively correlated with JRS total score (C). Abbreviations: BSP, blepharospasm; JRS, Jankovic Rating Scale; and MVOcC, ventromedial parietooccipital sulcus.


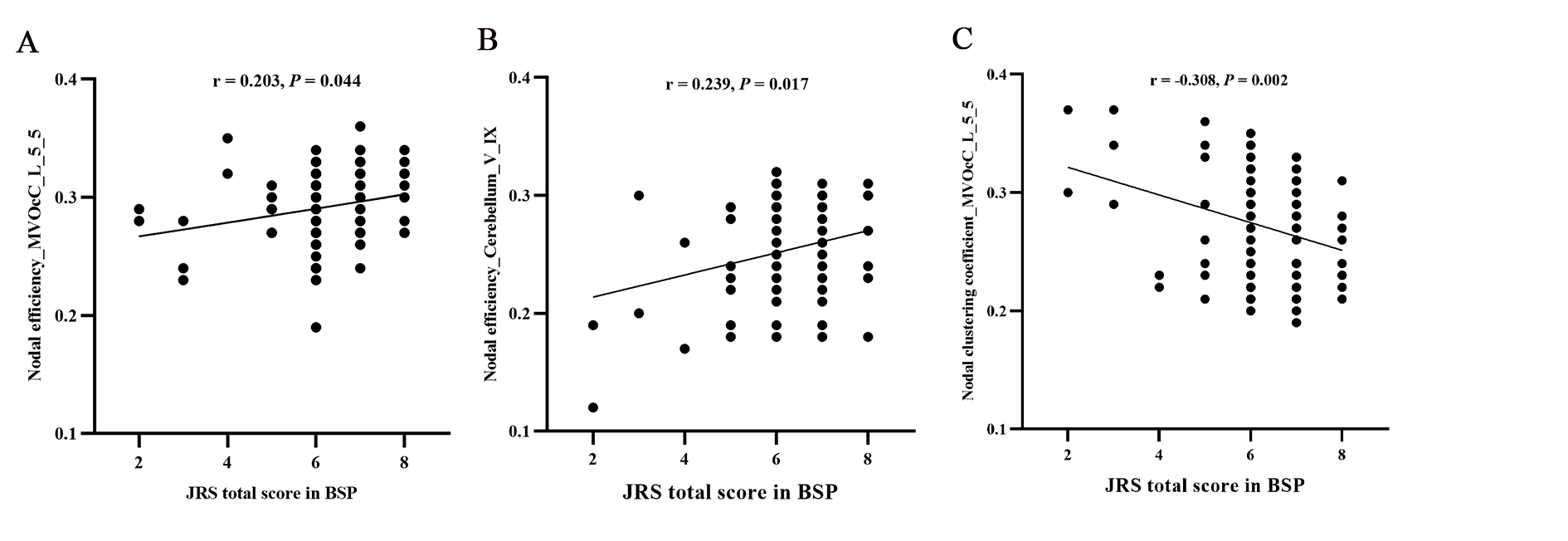


**Supplemental Tables**

**Table S1.** Cortical, subcortical, cerebellar regions and brain network of Brainnetome atlas.

1. Cortical and subcortical regions.

| **Gyrus** | **Label ID.L(R)** | **Left and Right Abbreviatio-ns** | **BN ID.L(R)** | **Region_name** |
| --- | --- | --- | --- | --- |
| SFG, Superior  Frontal Gyrus | 1(2) | SFG_L(R)_7_1 | 6(4) | medial area 8 |
|  | 3(4) | SFG_L(R)_7_2 | 7(6) | dorsolateral area 8 |
|  | 5(6) | SFG_L(R)_7_3 | 7(7) | lateral area 9 |
|  | 7(8) | SFG_L(R)_7_4 | 3(3) | dorsolateral area 6 |
|  | 9(10) | SFG_L(R)_7_5 | 2(2) | medial area 6 |
|  | 11(12) | SFG_L(R)_7_6 | 7(6) | medial area 9 |
|  | 13(14) | SFG_L(R)_7_7 | 7(7) | medial area 10 |
| MFG, Middle  Frontal Gyrus | 15(16) | MFG_L(R)_7_1 | 4(6) | dorsal area 9/46 |
|  | 17(18) | MFG_L(R)_7_2 | 6(6) | inferior frontal junction |
|  | 19(20) | MFG_L(R)_7_3 | 6(6) | area 46 |
|  | 21(22) | MFG_L(R)_7_4 | 6(6) | ventral area 9/46 |
|  | 23(24) | MFG_L(R)_7_5 | 7(6) | ventrolateral area 8 |
|  | 25(26) | MFG_L(R)_7_6 | 3(3) | ventrolateral area 6 |
|  | 27(28) | MFG_L(R)_7_7 | 5(6) | lateral area10 |
| IFG, Inferior  Frontal Gyrus | 29(30) | IFG_L(R)_6_1 | 6(3) | dorsal area 44 |
|  | 31(32) | IFG_L(R)_6_2 | 6(6) | inferior frontal sulcus |
|  | 33(34) | IFG_L(R)_6_3 | 7(7) | caudal area 45 |
|  | 35(36) | IFG_L(R)_6_4 | 7(6) | rostral area 45 |
|  | 37(38) | IFG_L(R)_6_5 | 4(4) | opercular area 44 |
|  | 39(40) | IFG_L(R)_6_6 | 4(4) | ventral area 44 |
| OrG, Orbital Gyrus | 41(42) | OrG_L(R)_6_1 | 7(7) | medial area 14 |
|  | 43(44) | OrG_L(R)_6_2 | 7(7) | orbital area 12/47 |
|  | 45(46) | OrG_L(R)_6_3 | 5(6) | lateral area 11 |
|  | 47(48) | OrG_L(R)_6_4 | 5(5) | medial area 11 |
|  | 49(50) | OrG_L(R)_6_5 | 5(5) | area 13 |
|  | 51(52) | OrG_L(R)_6_6 | 7(7) | lateral area 12/47 |
| PrG, Precentral  Gyrus | 53(54) | PrG_L(R)_6_1 | 2(2) | area 4 (head and face) |
|  | 55(56) | PrG_L(R)_6_2 | 3(3) | caudal dorsolateral area 6 |
|  | 57(58) | PrG_L(R)_6_3 | 2(2) | area 4 (upper limb) |
|  | 59(60) | PrG_L(R)_6_4 | 2(2) | area 4 (trunk region) |
|  | 61(62) | PrG_L(R)_6_5 | 4(4) | area 4 (tongue and larynx) |
|  | 63(64) | PrG_L(R)_6_6 | 3(3) | caudal ventrolateral area 6 |
| PCL, Paracentral  Lobule | 65(66) | PCL_L(R)_2_1 | 4(2) | area1/2/3 (lower limb) |
|  | 67(68) | PCL_L(R)_2_2 | 2(2) | area 4 (lower limb) |
| STG, Superior  Temporal Gyrus | 69(70) | STG_L(R)_6_1 | 5(5) | medial area 38 |
|  | 71(72) | STG_L(R)_6_2 | 2(2) | area 41/42 |
|  | 73(74) | STG_L(R)_6_3 | 2(2) | TE1.0 and TE1.2 |
|  | 75(76) | STG_L(R)_6_4 | 2(2) | caudal area 22 |
|  | 77(78) | STG_L(R)_6_5 | 5(5) | lateral area 38 |
|  | 79(80) | STG_L(R)_6_6 | 7(7) | rostral area 22 |
| MTG, Middle  Temporal Gyrus | 81(82) | MTG_L(R)_4_1 | 7(6) | caudal area 21 |
|  | 83(84) | MTG_L(R)_4_2 | 7(7) | rostral area 21 |
|  | 85(86) | MTG_L(R)_4_3 | 3(3) | dorsolateral area37 |
|  | 87(88) | MTG_L(R)_4_4 | 7(7) | anterior superior temporal  sulcus |
| ITG, Inferior  Temporal Gyrus | 89(90) | ITG_L(R)_7_1 | 5(5) | intermediate ventral area 20 |
|  | 91(92) | ITG_L(R)_7_2 | 3(3) | extreme lateroventral area37 |
|  | 93(94) | ITG_L(R)_7_3 | 5(5) | rostral area 20 |
|  | 95(96) | ITG_L(R)_7_4 | 7(5) | intermediate lateral area 20 |
|  | 97(98) | ITG_L(R)_7_5 | 3(3) | ventrolateral area 37 |
|  | 99(100) | ITG_L(R)_7_6 | 6(6) | caudolateral of area 20 |
|  | 101(102) | ITG_L(R)_7_7 | 5(5) | caudoventral of area 20 |
| FuG, Fusiform  Gyrus | 103(104) | FuG_L(R)_3_1 | 5(5) | rostroventral area 20 |
|  | 105(106) | FuG_L(R)_3_2 | 1(1) | medioventral area37 |
|  | 107(108) | FuG_L(R)_3_3 | 3(1) | lateroventral area37 |
| PhG,  Parahippocampal  Gyrus | 109(110) | PhG_L(R)_6_1 | 5(5) | rostral area 35/36 |
|  | 111(112) | PhG_L(R)_6_2 | 5(1) | caudal area 35/36 |
|  | 113(114) | PhG_L(R)_6_3 | 1(1) | lateral posterior  parahippocampal gyrus |
|  | 115(116) | PhG_L(R)_6_4 | 5(5) | area 28/34 |
|  | 117(118) | PhG_L(R)_6_5 | 5(5) | temporal agranular insular  cortex |
|  | 119(120) | PhG_L(R)_6_6 | 1(1) | medial posterior  parahippocampal gyrus |
| pSTS, Posterior  Superior  Temporal sulcus | 121(122) | pSTS_L(R)_2_1 | 7(7) | rostroposterior superior temporal sulcusal sulcus |
|  | 123(124) | pSTS_L(R)_2_2 | 4(4) | temporcaudoposterior superior  temporal sulcusal sulcus |
| SPL, Superior  Parietal Lobule | 125(126) | SPL_L(R)_5_1 | 3(3) | rostral area 7 |
|  | 127(128) | SPL_L(R)_5_2 | 3(3) | caudal area 7 |
|  | 129(130) | SPL_L(R)_5_3 | 3(3) | lateral area 5 |
|  | 131(132) | SPL_L(R)_5_4 | 2(2) | postcentral area 7 |
|  | 133(134) | SPL_L(R)_5_5 | 3(3) | intraparietal area 7 (hIP3) |
| IPL, Inferior  parietal lobule | 135(136) | IPL_L(R)_6_1 | 1(1) | caudal area 39 (PGp) |
|  | 137(138) | IPL_L(R)_6_2 | 6(6) | rostrodorsal area 39 (Hip3) |
|  | 139(140) | IPL_L(R)_6_3 | 3(3) | rostrodorsal area 40 (PFt) |
|  | 141(142) | IPL_L(R)_6_4 | 7(6) | caudal area 40 (PFm) |
|  | 143(144) | IPL_L(R)_6_5 | 3(7) | rostroventral area 39 (PGa) |
|  | 145(146) | IPL_L(R)_6_6 | 2(2) | rostroventral area 40 (PFop) |
| Pcun, Precuneus | 147(148) | PCun_L(R)_4_1 | 6(6) | medial area 7 (PEp) |
|  | 149(150) | PCun_L(R)_4_2 | 2(3) | medial area 5 (PEm) |
|  | 151(152) | PCun_L(R)_4_3 | 1(1) | dorsomedial parietooccipital sulcus |
|  | 153(154) | PCun_L(R)_4_4 | 7(7) | area 31 (Lc1) |
| PoG, Postcentral  gyrus | 155(156) | PoG_L(R)_4_1 | 2(2) | area 1/2/3 (upper limb, head and face) |
|  | 157(158) | PoG_L(R)_4_2 | 2(2) | area 1/2/3 (tongue and larynx) |
|  | 159(160) | PoG_L(R)_4_3 | 3(2) | area 2 |
|  | 161(162) | PoG_L(R)_4_4 | 2(2) | area1/2/3 (trunk) |
| INS, Insular gyrus | 163(164) | INS_L(R)_6_1 | 2(2) | hypergranular insula |
|  | 165(166) | INS_L(R)_6_2 | 8(6) | ventral agranular insula |
|  | 167(168) | INS_L(R)_6_3 | 4(4) | dorsal agranular insula |
|  | 169(170) | INS_L(R)_6_4 | 4(4) | ventral dysgranular and granular  insula |
|  | 171(172) | INS_L(R)_6_5 | 2(2) | dorsal granular insula |
|  | 173(174) | INS_L(R)_6_6 | 4(4) | dorsal dysgranular insula |
| CC, Cingulate  cortex | 175(176) | CG_L(R)_7_1 | 7(7) | dorsal area 23 |
|  | 177(178) | CG_L(R)_7_2 | 8(8) | rostroventral area 24 |
|  | 179(180) | CG_L(R)_7_3 | 7(4) | pregenual area 32 |
|  | 181(182) | CG_L(R)_7_4 | 7(1) | ventral area 23 |
|  | 183(184) | CG_L(R)_7_5 | 4(4) | caudodorsal area 24 |
|  | 185(186) | CG_L(R)_7_6 | 4(4) | caudal area 23 |
|  | 187(188) | CG_L(R)_7_7 | 7(7) | subgenual area 32 |
| MVOcC,  MedioVentralOccip  ital Cortex | 189(190) | MVOcC_L(R)_5_1 | 1(1) | caudal lingual gyrus |
|  | 191(192) | MVOcC_L(R)_5_2 | 1(1) | rostral cuneus gyrus |
|  | 193(194) | MVOcC_L(R)_5_3 | 1(1) | caudal cuneus gyrus |
|  | 195(196) | MVOcC_L(R)_5_4 | 1(1) | rostral lingual gyrus |
|  | 197(198) | MVOcC_L(R)_5_5 | 1(1) | ventromedial parietooccipital sulcus |
| LOcC, lateral  Occipital Cortex | 199(200) | LOcC_L(R)_4_1 | 1(1) | middle occipital gyrus |
|  | 201(202) | LOcC_L(R)_4_2 | 3(1) | area V5/MT+ |
|  | 203(204) | LOcC_L(R)_4_3 | 1(1) | occipital polar cortex |
|  | 205(206) | LOcC_L(R)_4_4 | 1(1) | inferior occipital gyrus |
|  | 207(208) | LOcC_L(R)_2_1 | 1(1) | medial superior occipital gyrus |
|  | 209(210) | LOcC_L(R)_2_2 | 1(1) | lateral superior occipital gyrus |
| Amyg, Amygdala | 211(212) | Amyg_L(R)_2_1 | 8(8) | medial amygdala |
|  | 213(214) | Amyg_L(R)_2_2 | 8(8) | lateral amygdala |
| Hipp,  Hippocampus | 215(216) | Hipp_L(R)_2_1 | 8(8) | rostral hippocampus |
|  | 217(218) | Hipp_L(R)_2_2 | 8(8) | caudal hippocampus |
| BG, Basal Ganglia | 219(220) | BG_L(R)_6_1 | 8(8) | ventral caudate |
|  | 221(222) | BG_L(R)_6_2 | 8(8) | globus pallidus |
|  | 223(224) | BG_L(R)_6_3 | 8(8) | nucleus accumbens |
|  | 225(226) | BG_L(R)_6_4 | 8(8) | ventromedial putamen |
|  | 227(228) | BG_L(R)_6_5 | 8(8) | dorsal caudate |
|  | 229(230) | BG_L(R)_6_6 | 8(8) | dorsolateral putamen |
| Tha, Thalamus | 231(232) | Tha_L(R)_8_1 | 8(8) | medial pre-frontal thalamus |
|  | 233(234) | Tha_L(R)_8_2 | 8(8) | pre-motor thalamus |
|  | 235(236) | Tha_L(R)_8_3 | 8(8) | sensory thalamus |
|  | 237(238) | Tha_L(R)_8_4 | 8(8) | rostral temporal thalamus |
|  | 239(240) | Tha_L(R)_8_5 | 8(8) | posterior parietal thalamus |
|  | 241(242) | Tha_L(R)_8_6 | 8(8) | occipital thalamus |
|  | 243(244) | Tha_L(R)_8_7 | 8(8) | caudal temporal thalamus |
|  | 245(246) | Tha_L(R)_8_8 | 8(8) | lateral pre-frontal thalamus |

Abbreviations: BN, brain network; Labels ID.L, Labels ID.Left; Labels ID.R, Labels ID.Right.

1. Cerebellar regions of Brainnetome atlas.

| **Gyrus** | **Labels ID.L** | **Labels ID.V** | **Labels ID.R** | **Left(Right) and Vermis Region** | **BN** |
| --- | --- | --- | --- | --- | --- |
| Cerebellum | 247 | - | 248 | Cerebellum_L(R)_I-IV | 9 |
|  | 249 | - | 250 | Cerebellum_L(R)_V | 9 |
|  | 251 | 252 | 253 | Cerebellum_L(V)(R)_VI | 9 |
|  | 254 | 255 | 256 | Cerebellum_L(V)(R)_Crus_I | 9 |
|  | 257 | 258 | 259 | Cerebellum_L(V)(R)_Crus_II | 9 |
|  | 260 | 261 | 262 | Cerebellum_L(V)(R)_VIIb | 9 |
|  | 263 | 264 | 265 | Cerebellum_L(V)(R)_VIIIa | 9 |
|  | 266 | 267 | 268 | Cerebellum_L(V)(R)_VIIIb | 9 |
|  | 269 | 270 | 271 | Cerebellum_L(V)(R)_IX | 9 |
|  | 272 | 273 | 274 | Cerebellum_L(V)(R)_X | 9 |

Abbreviations: Labels ID.V, Labels ID. Vermis.

1. Brain network (BN).

| **ID** | **Network name** |
| --- | --- |
| 1 | Visual network |
| 2 | Somatomotor network |
| 3 | Dorsal Attention network |
| 4 | Ventral Attention network |
| 5 | Limbic network |
| 6 | Frontoparietal network |
| 7 | Default mode network |
| 8 | Subcortical network |
| 9 | Cerebellar network |

**Table S2.** Comparison of global network properties between patients with BSP and HCs.

|  | **BSP**  **(n = 102; mean ± SD)** | **HCs**  **(n = 160; mean ± SD)** | ***T*** | **FDR corrected *P*** |
| --- | --- | --- | --- | --- |
| **a) BNA-274** | | | | |
| E_loc_ | 0.356 ± 0.005 | 0.358± 0.005 | -3.21 | 0.002 ^*^ |
| E_g_ | 0.268±0.005 | 0.267±0.005 | 2.25 | 0.025 ^*^ |
| C_p_ | 0.275 ± 0.011 | 0.279 ± 0.011 | -2.72 | 0.007 ^*^ |
| L_p_ | 0.793 ± 0.024 | 0.797 ± 0.022 | -1.67 | 0.096 |
| **b) AAL_116** | | | | |
| E_loc_ | 0.346 ± 0.007 | 0.348 ± 0.007 | -1.68 | 0.095 |
| E_g_ | 0.256 ± 0.008 | 0.254± 0.008 | 2.57 | 0.011 ^*^ |
| C_p_ | 0.277 ± 0.009 | 0.280 ± 0.010 | -2.91 | 0.004 ^*^ |
| L_p_ | 0.867 ± 0.046 | 0.878 ± 0.047 | -2.31 | 0.022 ^*^ |
| **c) rSchaefer-100** | | | | |
| E_loc_ | 0.348 ± 0.006 | 0.349 ± 0.006 | -1.39 | 0.167 |
| E_g_ | 0.260 ± 0.007 | 0.259 ± 0.007 | 1.86 | 0.065 |
| C_p_ | 0.274 ± 0.009 | 0.276 ± 0.010 | -1.98 | 0.050 |
| L_p_ | 0.840 ± 0.038 | 0.845 ± 0.037 | -1.46 | 0.146 |
| **d) rSchaefer-300** | | | | |
| E_loc_ | 0.356 ± 0.005 | 0.359 ± 0.005 | -3.12 | 0.002 ^*^ |
| E_g_ | 0.269 ± 0.005 | 0.268 ± 0.005 | 2.09 | 0.038 ^*^ |
| C_p_ | 0.275 ± 0.012 | 0.279 ± 0.011 | -2.81 | 0.005 ^*^ |
| L_p_ | 0.788 ± 0.024 | 0.793 ± 0.228 | -1.73 | 0.085 |
| **e) rSchaefer-500** | | | | |
| E_loc_ | 0.358 ± 0.005 | 0.360 ± 0.005 | -3.50 | <0.001 ^*^ |
| E_g_ | 0.272 ± 0.005 | 0.271 ± 0.004 | 2.21 | 0.028 ^*^ |
| C_p_ | 0.274 ± 0.013 | 0.279 ± 0.012 | -2.99 | 0.003 ^*^ |
| L_p_ | 0.773 ± 0.020 | 0.777 ± 0.018 | -1.90 | 0.058 |
| **f) rSchaefer-700** | | | | |
| E_loc_ | 0.359 ± 0.006 | 0.362 ± 0.006 | -3.66 | <0.001 ^*^ |
| E_g_ | 0.274 ± 0.004 | 0.272 ± 0.004 | 2.32 | 0.021 ^*^ |
| C_p_ | 0.274 ± 0.014 | 0.279 ± 0.013 | -3.17 | 0.002 ^*^ |
| L_p_ | 0.767 ± 0.018 | 0.771 ± 0.016 | -1.89 | 0.060 |

Abbreviations: BSP, blepharospasm; C_p_, clustering coefficient; E_g_, global efficiency; E_loc_, local efficiency; HCs, healthy controls; L_p_, characteristic path length. **P* < 0.05.

**Table S3.** Comparison of global network properties between patients with BOD and HCs.

|  | **BOD**  **(n = 43; mean ± SD)** | **HCs**  **(n = 160; mean ± SD)** | ***T*** | **FDR corrected *P*** |
| --- | --- | --- | --- | --- |
| **a) BNA-274** | | | | |
| E_loc_ | 0.356 ± 0.006 | 0.358 ± 0.005 | -2.53 | 0.012 ^*^ |
| E_g_ | 0.268 ± 0.005 | 0.267 ± 0.005 | 2.34 | 0.020 ^*^ |
| C_p_ | 0.274 ± 0.012 | 0.279 ± 0.011 | -2.78 | 0.006 ^*^ |
| L_p_ | 0.7924 ± 0.0241 | 0.797 ± 0.022 | -2.09 | 0.038 ^*^ |
| **b) AAL_116** | | | | |
| E_loc_ | 0.347±0.010 | 0.358 ± 0.007 | -0.66 | 0.511 |
| E_g_ | 0.356 ± 0.006 | 0.358 ± 0.005 | 1.58 | 0.116 |
| C_p_ | 0.278 ± 0.010 | 0.280 ± 0.010 | -3.33 | 0.001 ^*^ |
| L_p_ | 0.876 ± 0.052 | 0.878 ± 0.047 | -1.15 | 0.254 |
| **c) rSchaefer-100** | | | | |
| E_loc_ | 0.348 ± 0.008 | 0.349 ± 0.006 | -0.87 | 0.383 |
| E_g_ | 0.259 ± 0.007 | 0.259± 0.066 | 1.54 | 0.126 |
| C_p_ | 0.274 ± 0.009 | 0.278 ± 0.010 | -1.49 | 0.137 |
| L_p_ | 0.845 ± 0.040 | 0.845 ± 0.037 | -0.91 | 0.365 |
| **d) rSchaefer-300** | | | | |
| E_loc_ | 0.356 ± 0.006 | 0.359 ± 0.005 | -2.38 | 0.018 ^*^ |
| E_g_ | 0.269 ± 0.004 | 0.268 ± 0.005 | 1.92 | 0.057 |
| C_p_ | 0.275 ± 0.011 | 0.279 ± 0.011 | -2.81 | 0.005 ^*^ |
| L_p_ | 0.789 ± 0.022 | 0.793±0.023 | -0.91 | 0.365 |
| **e) rSchaefer-500** | | | | |
| E_loc_ | 0.358 ± 0.006 | 0.360 ± 0.005 | -3.05 | 0.003 ^*^ |
| E_g_ | 0.272 ± 0.004 | 0.271 ± 0.004 | 2.44 | 0.016 ^*^ |
| C_p_ | 0.273 ± 0.130 | 0.279 ± 0.012 | -2.89 | 0.004 ^*^ |
| L_p_ | 0.773 ± 0.159 | 0.777 ± 0.018 | -2.19 | 0.047 ^*^ |
| **f) rSchaefer-700** | | | | |
| E_loc_ | 0.359 ± 0.007 | 0.362 ± 0.006 | -3.15 | 0.002 ^*^ |
| E_g_ | 0.273 ± 0.003 | 0.272 ± 0.004 | 2.22 | 0.028 ^*^ |
| C_p_ | 0.273 ± 0.014 | 0.279 ± 0.013 | -3.00 | 0.003 ^*^ |
| L_p_ | 0.767 ± 0.014 | 0.771 ± 0.0164 | -2.01 | 0.046 ^*^ |

Abbreviations: BOD, blepharospasm-oromandibular dystonia; C_p_, clustering coefficient; E_g_, global efficiency; E_loc_, local efficiency; and L_p_, characteristic path length. **P* < 0.05.

**Table S4.** Comparison of global network properties between patients with CD and HCs.

|  | **CD**  **(n = 56; mean ± SD)** | **HCs**  **(n = 160; mean ± SD)** | ***T*** | **FDR corrected *P*** |
| --- | --- | --- | --- | --- |
| **a) BNA-274** | | | | |
| E_loc_ | 0.357 ± 0.004 | 0.358 ± 0.005 | -1.52 | 0.130 |
| E_g_ | 0.269 ± 0.008 | 0.267 ± 0.005 | 1.04 | 0.301 |
| C_p_ | 0.276 ± 0.009 | 0.279 ± 0.011 | -1.56 | 0.249 |
| L_p_ | 0.789 ± 0.022 | 0.797 ± 0.022 | -1.12 | 0.263 |
| **b) AAL_116** | | | | |
| E_loc_ | 0.348 ± 0.006 | 0.348 ± 0.007 | -1.12 | 0.263 |
| E_g_ | 0.257 ± 0.008 | 0.254 ± 0.008 | 1.20 | 0.232 |
| C_p_ | 0.277 ± 0.009 | 0.280 ± 0.010 | -1.48 | 0.140 |
| L_p_ | 0.861 ± 0.043 | 0.878 ± 0.047 | -1.40 | 0.162 |
| **c) rSchaefer-100** | | | | |
| E_loc_ | 0.350 ± 0.005 | 0.349 ± 0.006 | 0.26 | 0.793 |
| E_g_ | 0.261 ± 0.006 | 0.259 ± 0.007 | 1.44 | 0.152 |
| C_p_ | 0.274 ± 0.009 | 0.276 ± 0.010 | -3.26 | 0.001 ^*^ |
| L_p_ | 0.832 ± 0.031 | 0.845 ± 0.037 | -1.366 | 0.174 |
| **d) rSchaefer-300** | | | | |
| E_loc_ | 0.358 ± 0.005 | 0.359 ± 0.005 | -2.66 | 0.790 |
| E_g_ | 0.270 ± 0.004 | 0.268 ± 0.008 | 1.47 | 0.144 |
| C_p_ | 0.276 ± 0.010 | 0.279 ± 0.011 | -0.72 | 0.471 |
| L_p_ | 0.785 ± 0.018 | 0.793 ± 0.023 | -1.465 | 0.144 |
| **e) rSchaefer-500** | | | | |
| E_loc_ | 0.359 ± 0.005 | 0.360 ± 0.005 | -0.99 | 0.323 |
| E_g_ | 0.273 ± 0.003 | 0.271 ± 0.004 | 1.58 | 0.114 |
| C_p_ | 0.275 ± 0.011 | 0.2785 ± 0.012 | -1.08 | 0.283 |
| L_p_ | 0.771 ± 0.014 | 0.777 ± 0.018 | -1.58 | 0.115 |
| **f) rSchaefer-700** | | | | |
| E_loc_ | 0.360 ± 0.005 | 0.362 ± 0.006 | -1.11 | 0.267 |
| E_g_ | 0.274 ± 0.003 | 0.272 ± 0.004 | 1.54 | 0.126 |
| C_p_ | 0.276 ± 0.1117 | 0.279 ± 0.129 | -1.14 | 0.257 |
| L_p_ | 0.766 ± 0.012 | 0.771 ± 0.016 | -1.46 | 0.147 |

Abbreviations: CD, cervical dystonia; C_p_, clustering coefficient; E_g_, global efficiency; E_loc_, local efficiency; and L_p_, characteristic path length. **P* < 0.05.

**Table S5.** Differences in degree centrality, nodal efficiency, nodal clustering coefficient, and nodal local efficiency based on BNA-274 atlas between patients with idiopathic dystonia and HCs.

| **Labels** | **Region name** | **Abbreviations** | **BN** | **Dystonia (mean ± SD)** | **HCs (mean ± SD)** | ***T*** | **FDR**  **correct *P*** |
| --- | --- | --- | --- | --- | --- | --- | --- |
| 1. **Degree centrality (Dystonia < HCs)** | | | | | | | |
| 46 | lateral area 11 | OrG_R_6_3 | 7 | 34.04 ± 13.82 | 38.25 ± 14.53 | -3.13 | <0.05 |
| 145 | rostroventral area 40 (PFop) | IPL_L_6_6 | 2 | 38.34 ± 11.87 | 42.64 ± 10.81 | -3.49 | <0.05 |
| 163 | hypergranular insula | INS_L_6_1 | 2 | 36.11 ± 13.40 | 41.41 ± 11.73 | -3.78 | <0.05 |
| 171 | dorsal granular insula | INS_L_6_5 | 2 | 31.40 ± 12.36 | 36.06 ± 11.89 | -3.61 | <0.05 |
| 1. **Degree centrality (Dystonia > HCs)** | | | | | | | |
| 231 | medial pre-frontal thalamus | Tha_L_8_1 | 8 | 37.09 ± 16.37 | 30.21 ± 15.60 | 4.31 | <0.05 |
| 232 |  | Tha_R_8_1 | 8 | 38.21 ± 17.19 | 31.43 ± 15.60 | 4.20 | <0.05 |
| 237 | rostral temporal thalamus | Tha_L_8_4 | 8 | 29.56 ± 14.18 | 23.59 ± 13.35 | 4.31 | <0.05 |
| 238 |  | Tha_R_8_4 | 8 | 24.02 ± 14.07 | 19.20 ± 12.72 | 3.66 | <0.05 |
| 245 | lateral pre-frontal thalamus | Tha_L_8_8 | 8 | 43.84 ± 16.99 | 37.55 ± 15.68 | 3.72 | <0.05 |
| 246 |  | Tha_R_8_8 | 8 | 43.60 ± 17.35 | 36.82 ± 16.51 | 3.90 | <0.05 |
| 268 |  | Cerebellum_R_VIIIb | 9 | 29.12 ± 14.28 | 24.85 ± 14.28 | 3.14 | <0.05 |
| 270 |  | Cerebellum_V_IX | 9 | 28.22 ± 14.89 | 22.83 ± 13.72 | 3.93 | <0.05 |
| 1. **Nodal efficiency (Dystonia < HCs)** | | | | | | | |
| 145 | rostroventral area 40 (PFop) | IPL_L_6_6 | 2 | 0.279 ± 0.027 | 0.290 ± 0.023 | -3.63 | <0.05 |
| 163 | hypergranular insula | INS_L_6_1 | 2 | 0.275 ± 0.031 | 0.286 ± 0.027 | -3.53 | <0.05 |
| 171 | dorsal granular insula | INS_L_6_5 | 2 | 0.263 ± 0.030 | 0.273 ± 0.279 | -3.37 | <0.05 |
| 1. **Nodal efficiency (Dystonia > HCs)** | | | | | | | |
| 227 | ventromedial putamen | BG_L_6_5 | 8 | 0.222 ± 0.049 | 0.204 ± 0.054 | 3.59 | <0.05 |
| 228 |  | BG_R_6_5 | 8 | 0.225 ± 0.051 | 0.208 ± 0.059 | 3.42 | <0.05 |
| 231 | medial pre-frontal thalamus | Tha_L_8_1 | 8 | 0.273 ± 0.045 | 0.256 ± 0.043 | 3.89 | <0.05 |
| 232 |  | Tha_R_8_1 | 8 | 0.276 ± 0.047 | 0.259 ± 0.042 | 3.65 | <0.05 |
| 237 | rostral temporal thalamus | Tha_L_8_4 | 8 | 0.256 ± 0.042 | 0.237 ± 0.042 | 4.17 | <0.05 |
| 238 |  | Tha_R_8_4 | 8 | 0.241 ± 0.044 | 0.225 ± 0.425 | 3.66 | <0.05 |
| 245 | lateral pre-frontal thalamus | Tha_L_8_8 | 8 | 0.289 ± 0.447 | 0.275 ± 0.039 | 3.21 | <0.05 |
| 246 |  | Tha_R_8_8 | 8 | 0.288 ± 0.047 | 0.273 ± 0.041 | 3.29 | <0.05 |
| 270 |  | Cerebellum_V_IX | 9 | 0.251 ± 0.044 | 0.236 ± 0.044 | 3.70 | <0.05 |
| 1. **Nodal clustering coefficient (Dystonia < HCs)** | | | | | | | |
| 54 | area 4 (head and face) | PrG_R_6_1 | 2 | 0.309 ± 0.536 | 0.328 ± 0.048 | -3.26 | <0.05 |
| 107 | lateroventral area37 | FuG_L_3_3 | 3 | 0.261 ± 0.037 | 0.276 ± 0.039 | -3.46 | <0.05 |
| 197 | ventromedial parietooccipital sulcus | MVOcC_L_5_5 | 1 | 0.277 ± 0.046 | 0.293 ± 0.417 | -3.43 | <0.05 |
| 207 | medial superior occipital gyrus | LOcC_L_2_1 | 1 | 0.311 ± 0.045 | 0.328 ± 0.048 | -3.36 | <0.05 |
| 237 | rostral temporal thalamus | Tha_L_8_4 | 8 | 0.279 ± 0.038 | 0.292 ± 0.407 | -3.34 | <0.05 |
| 238 |  | Tha_R_8_4 | 8 | 0.310 ± 0.051 | 0.327 ± 0.049 | -3.34 | <0.05 |
| 245 | lateral pre-frontal thalamus | Tha_L_8_8 | 8 | 0.246 ± 0.034 | 0.261 ± 0.372 | -4.02 | <0.05 |
| 246 |  | Tha_R_8_8 | 8 | 0.245 ± 0.035 | 0.258 ± 0.039 | -3.30 | <0.05 |
| 1. **Nodal local efficiency (Dystonia < HCs)** | | | | | | | |
| 122 | rostroposterior superior | pSTS_R_2_1 | 7 | 0.363 ± 0.027 | 0.371 ± 0,197 | -3.68 | <0.05 |
| 197 | ventromedial parietooccipital sulcus | MVOcC_L_5_5 | 1 | 0.363 ± 0.024 | 0.371 ± 0.022 | -3.42 | <0.05 |
| 207 | medial superior occipital gyrus | LOcC_L_2_1 | 1 | 0.380 ± 0.023 | 0.388 ± 0.025 | -3.32 | <0.05 |
| 245 | lateral pre-frontal thalamus | Tha_L_8_8 | 8 | 0.346 ± 0.019 | 0.353 ± 0.021 | -3.69 | <0.05 |

Abbreviations: BNA-274, Brainnetome atlas with 274 brain regions; and HCs, healthy controls.

**Table S6.** Differences in degree centrality, nodal efficiency, nodal clustering coefficient, and nodal local efficiency based on BNA-274 atlas between patients with BSP and HCs.

| **Labels** | **Region name** | **Abbreviations** | **BN** | **BSP (n = 102; mean ± SD)** | **HCs (n = 160; mean ± SD)** | ***T*** | **FDR**  **correct *P*** |
| --- | --- | --- | --- | --- | --- | --- | --- |
| 1. **Degree centrality** **(BSP < HCs)** | | | | | | | |
| 163 | hypergranular insula | INS_L_6_1 | 2 | 34.831 ± 12.489 | 41.41 ± 11.73 | -3.52 | <0.05 |
| 1. **Degree centrality (BSP > HCs)** | | | | | | | |
| 231 | medial pre-frontal thalamus | Tha_L_8_1 | 8 | 37.687 ± 15.369 | 30.214 ± 15.597 | 3.87 | <0.05 |
| 232 |  | Tha_R_8_1 | 8 | 39.020 ± 16.981 | 31.426 ± 15.602 | 3.83 | <0.05 |
| 237 | rostral temporal thalamus | Tha_L_8_4 | 8 | 30.231 ± 13.792 | 23.594 ± 13.352 | 3.86 | <0.05 |
| 238 |  | Tha_R_8_4 | 8 | 25.222 ± 14.440 | 19.205 ± 12.719 | 3.75 | <0.05 |
| 245 | lateral pre-frontal thalamus | Tha_L_8_8 | 8 | 44.503 ± 15.833 | 37.552 ± 16.682 | 3.43 | <0.05 |
| 246 |  | Tha_R_8_8 | 8 | 44.575 ± 17.236 | 36.819 ± 16.514 | 3.51 | <0.05 |
| 1. **Nodal efficiency (BSP > HCs)** | | | | | | | |
| 197 | ventromedial parietooccipital sulcus | MVOcC_L_5_5 | 1 | 0.291 ± 0.031 | 0.279 ± 0.028 | 3.18 | <0.05 |
| 227 | ventromedial putamen | BG_L_6_5 | 8 | 0.225 ± 0.051 | 0.204 ± 0.054 | 3.38 | <0.05 |
| 231 | medial pre-frontal thalamus | Tha_L_8_1 | 8 | 0.276 ± 0.041 | 0.256 ± 0.043 | 3.75 | <0.05 |
| 232 |  | Tha_R_8_1 | 8 | 0.278 ± 0.045 | 0.260 ± 0.042 | 3.47 | <0.05 |
| 237 | rostral temporal thalamus | Tha_L_8_4 | 8 | 0.258 ± 0.039 | 0.238 ± 0.043 | 3.93 | <0.05 |
| 238 |  | Tha_R_8_4 | 8 | 0.245 ± 0.043 | 0.225 ± 0.042 | 3.85 | <0.05 |
| 245 | lateral pre-frontal thalamus | Tha_L_8_8 | 8 | 0.292 ± 0.040 | 0.275 ± 0.040 | 3.23 | <0.05 |
| 246 |  | Tha_R_8_8 | 8 | 0.291 ± 0.044 | 0.273 ± 0.041 | 3.23 | <0.05 |
| 258 |  | Cerebellum_V_Crus_II | 9 | 0.281 ± 0.029 | 0.269 ± 0.034 | 3.20 | <0.05 |
| 270 |  | Cerebellum_V_IX | 9 | 0.253 ± 0.043 | 0.236 ± 0.044 | 3.38 | <0.05 |
| 1. **Nodal clustering coefficient (BSP < HCs)** | | | | | | | |
| 166 | ventral agranular insula | INS_LR_6_2 | 6 | 0.260 ± 0.058 | 0.283 ± 0.044 | -3.67 | <0.05 |
| 197 | ventromedial parietooccipital sulcus | MVOcC_L_5_5 | 1 | 0.273 ± 0.045 | 0.293 ± 0.042 | -3.61 | <0.05 |
| 198 |  | MVOcC_R_5_5 | 1 | 0.259 ± 0.032 | 0.276 ± 0.037 | -3.56 | <0.05 |
| 238 | rostral temporal thalamus | Tha_R_8_4 | 8 | 0.304 ± 0.053 | 0.327 ± 0.049 | -4.00 | <0.05 |
| 245 | lateral pre-frontal thalamus | Tha_L_8_8 | 8 | 0.243 ± 0.032 | 0.261 ± 0.037 | -4.00 | <0.05 |
| 1. **Nodal local efficiency (BSP < HCs)** | | | | | | | |
| 197 | ventromedial parietooccipital sulcus | MVOcC_L_5_5 | 1 | 0.360 ± 0.024 | 0.371 ± 0.022 | -3.65 | <0.05 |
| 238 | rostral temporal thalamus | Tha_R_8_4 | 8 | 0.373 ± 0.037 | 0.386 ± 0.027 | -3.77 | <0.05 |
| 245 | lateral pre-frontal thalamus | Tha_L_8_8 | 8 | 0.344 ± 0.018 | 0.353 ± 0.021 | -3.76 | <0.05 |

Abbreviations: BNA-274, Brainnetome atlas with 274 brain regions; BSP, blepharospasm; and HCs, healthy controls.

**Table S7.** Differences in degree centrality based on BNA-274 atlas between patients with CD and HCs.

| **Labels** | **Region name** | **Abbreviations** | **BN** | **CD (n = 56; mean ± SD)** | **HCs (n = 160; mean ± SD)** | ***T*** | **FDR**  **correct *P*** |
| --- | --- | --- | --- | --- | --- | --- | --- |
| 1. **Degree Centrality (CD > HCs)** | | | | | | | |
| 4 | dorsolateral area 8 | SFG_R_7_2 | 6 | 30.971 ± 10.486 | 26.493 ± 10.891 | 3.56 | <0.05 |
| 231 | medial pre-frontal thalamus | Tha_L_8_1 | 8 | 40.214 ± 16.124 | 30.229 ± 15.602 | 3.49 | <0.05 |
| 232 |  | Tha_R_8_1 | 8 | 42.251 ± 15.834 | 31.437 ± 15.602 | 3.98 | <0.05 |
| 233 | pre-motor thalamus | Tha_L_8_2 | 8 | 38.533 ± 12.779 | 30.972 ± 13.501 | 3.52 | <0.05 |
| 246 | lateral pre-frontal thalamus | Tha_R_8_8 | 8 | 46.355 ± 14.974 | 36.832 ± 16.515 | 3.45 | <0.05 |
| 268 |  | Cerebellum_R_VIIIb | 9 | 32.253 ± 13.606 | 24.856 ± 14.030 | 3.31 | <0.05 |
| 270 |  | Cerebellum_V_IX | 9 | 31.284 ± 13.7774 | 22.841 ± 13.720 | 3.86 | <0.05 |

Abbreviations: BNA-274, Brainnetome atlas with 274 brain regions; CD, cervical dystonia; and HCs, healthy controls.

**Table S8.** Distributions of hub regions of degree centrality based on BNA-274 atlas for patients with idiopathic dystonia and HCs.

| **Dystonia specific labels** | **Region name** | **Abbreviations** | **BN** | **HCs specific labels** | **Region name** | **Abbreviations** | **BN** |
| --- | --- | --- | --- | --- | --- | --- | --- |
| 6 | lateral area 9 | SFG_R_7_3 | 7 | 4 | dorsolateral area 8 | SFG_R_7_2 | 6 |
| 14 | medial area 10 | SFG_R_7_7 | 7 | 22 | ventral area 9/46 | MFG_R_7_4 | 6 |
| 15 | dorsal area 9-46 | MFG_L_7_1 | 4 | 121 | rostroposterior superior | pSTS_L_2_1 | 7 |
| 21 | ventral area 9/46 | MFG_L_7_4 | 6 | 137 | rostrodorsal area 39 (Hip3) | IPL_L_6_2 | 6 |
| 29 | dorsal area 44 | IFG_L_6_1 | 6 | 219 | ventral caudate | BG_L_6_1 | 8 |
| 32 | inferior frontal sulcus | IFG_R_6_2 | 6 | 228 | dorsal caudate | BG_R_6_5 | 8 |
| 37 | opercular area 44 | IFG_L_6_5 | 4 | 242 | occipital thalamus | Tha_R_8_6 | 8 |
| 39 | ventral area 44 | IFG_L_6_6 | 4 | 244 | caudal temporal thalamus | Tha_R_8_7 | 8 |
| 40 |  | IFG_R_6_6 | 4 | 260 |  | Cerebellum_L_VIIb | 9 |
| 52 | lateral area 12/47 | OrG_R_6_6 | 7 | 272 |  | Cerebellum_L_X | 9 |
| 64 | caudal ventrolateral area 6 | PrG_R_6_6 | 3 |  |  |  |  |
| 116 | area 28/34 | PhG_R_6_4 | 5 |  |  |  |  |
| 127 | caudal area 7 | SPL_L_5_2 | 3 |  |  |  |  |
| 133 | intraparietal area 7 (hIP3) | SPL_L_5_5 | 3 |  |  |  |  |
| 141 | caudal area 40 (PFm) | IPL_L_6_4 | 7 |  |  |  |  |
| 165 | ventral agranular insula | INS_L_6_2 | 8 |  |  |  |  |
| 168 | dorsal agranular insula | INS_R_6_3 | 4 |  |  |  |  |
| 180 | pregenual area 32 | CG_R_7_3 | 4 |  |  |  |  |
| 195 | rostral lingual gyrus | MVOcC_L_5_4 | 1 |  |  |  |  |
| 199 | middle occipital gyrus | LOcC_L_4_1 | 1 |  |  |  |  |
| 212 | medial amygdala | Amyg_R_2_1 | 8 |  |  |  |  |
| 220 | ventral caudate | BG_R_6_1 | 8 |  |  |  |  |
| 263 |  | Cerebellum_L_VIIIa | 9 |  |  |  |  |
| 269 |  | Cerebellum_L_IX | 9 |  |  |  |  |
| 273 |  | Cerebellum_V_X | 9 |  |  |  |  |
| **Shared labels** | **Region name** | **Abbreviations** | **BN** | **Shared labels** | **Region name** | **Abbreviations** | **BN** |
| 13 | medial area 10 | SFG_L_7_7 | 7 | 227 | dorsal caudate | BG_L_6_5 | 8 |
| 42 | medial area 14 | OrG_R_6_1 | 7 | 238 | rostral temporal thalamus | Tha_R_8_4 | 8 |
| 98 | ventrolateral area 37 | ITG_R_7_5 | 3 | 243 | caudal temporal thalamus | Tha_R_8_7 | 8 |
| 114 | lateral posterior  parahippocampal gyrus | PhG_R_6_3 | 1 | 272 |  | Cerebellum_L_X | 9 |
| 118 | temporal agranular insular  cortex | PhG_R_6_5 | 5 |  |  |  |  |

Abbreviations: BNA-274, Brainnetome atlas with 274 brain regions; and HCs, healthy controls.

**Table S9.** Distributions of hub regions of degree centrality based on BNA-274 atlas for patients with BSP (n = 102) and HCs (n = 160).

| **BSP specific labels** | **Region name** | **Abbreviations** | **BN** | **HCs specific labels** | **Region name** | **Abbreviations** | **BN** |
| --- | --- | --- | --- | --- | --- | --- | --- |
| 6 | lateral area 9 | SFG_R_7_3 | 7 | 4 | dorsolateral area 8 | SFG_R_7_2 | 6 |
| 15 | dorsal area 9-46 | MFG_L_7_1 | 4 | 22 | ventral area 9/46 | MFG_R_7_4 | 6 |
| 32 | inferior frontal sulcus | IFG_R_6_2 | 6 | 121 | rostroposterior superior | pSTS_L_2_1 | 7 |
| 40 | ventral area 44 | IFG_L_6_6 | 4 | 137 | rostrodorsal area 39 (Hip3) | IPL_L_6_2 | 6 |
| 52 | lateral area 12/47 | OrG_R_6_6 | 7 | 219 | ventral caudate | BG_L_6_1 | 8 |
| 64 | caudal ventrolateral area 6 | PrG_R_6_6 | 3 | 228 | dorsal caudate | BG_R_6_5 | 8 |
| 132 | postcentral area 7 | SPL_R_5_4 | 2 | 242 | occipital thalamus | Tha_R_8_6 | 8 |
| 133 | intraparietal area 7 (hIP3) | SPL_L_5_5 | 3 | 244 | caudal temporal thalamus | Tha_R_8_7 | 8 |
| 165 | ventral agranular insula | INS_L_6_2 | 8 | 260 |  | Cerebellum_L_VII | 9 |
| 180 | pregenual area 32 | CG_R_7_3 | 4 | 272 |  | Cerebellum_L_X | 9 |
| 273 |  | Cerebellum_V_X | 9 | 13 | medial area 10 | SFG_L_7_7 | 7 |
|  |  |  |  | 42 | medial area 14 | OrG_R_6_1 | 7 |
|  |  |  |  | 98 | ventrolateral area 37 | ITG_R_7_5 | 3 |
|  |  |  |  | 227 | dorsal caudate | BG_L_6_5 | 8 |
|  |  |  |  | 238 | rostral temporal thalamus | Tha_R_8_4 | 8 |
|  |  |  |  | 243 | caudal temporal thalamus | Tha_R_8_7 | 8 |
|  |  |  |  | 272 |  | Cerebellum_L_X | 9 |
| **Shared labels** | **Region name** | **Abbreviations** | **BN** | **Shared labels** | **Region name** | **Abbreviations** | **BN** |
| 114 | lateral posterior  parahippocampal gyrus | PhG_R_6_3 | 1 | 118 | temporal agranular insular  cortex | PhG_R_6_5 | 5 |

Abbreviations: BNA-274, Brainnetome atlas with 274 brain regions; BSP, blepharospasm; and HCs, healthy controls.

**Table S10.** Subject demographics between subgroups of patients with idiopathic dystonia (BSP, BOD, and CD) and their age-matched healthy controls, respectively.

|  | **BSP *v.s.* age-matched HCs (n = 102)** | | | **BOD *v.s.* age-matched HCs (n = 43)** | | | **CD *v.s.* age-matched HCs (n = 56)** | | |
| --- | --- | --- | --- | --- | --- | --- | --- | --- | --- |
|  | BSP | HCs | *P* value | BOD | HCs | *P* value | CD | HCs | *P* value |
| Sex (F/M) ^a^ | 71/31 | 61/41 | 0.143 | 29/14 | 28/15 | 0.820 | 30/26 | 31/25 | 0.850 |
| Age ^b^ | 53.69 ± 9.01 | 53.06 ± 10.54 | 0.643 | 56.25 ± 10.94 | 57.35 ± 10.15 | 0.611 | 40.33±11.72 | 43.98 ± 13.10 | 0.123 |
| FD ^b^ | 0.16 ± 0.09 | 0.18 ± 0.07 | 0.429 | 0.16 ± 0.07 | 0.17 ± 0.07 | 0.967 | 0.16±0.07 | 0.17 ± 0.07 | 0.982 |
| HAMA ^c^ | 4.0 (0-22) | 2.0 (0-19) | < 0.001 | 7.0 (0-21) | 2.5 (0-10) | < 0.001 | 6.0 (0-23) | 3.0 (0-19) | < 0.001 |
| HAMD ^c^ | 3.0 (0-24) | 1.0 (0-13) | < 0.001 | 5.0 (0-19) | 2.0 (0-8) | 0.015 | 5.0 (0-19) | 1.0 (0-13) | < 0.001 |

Abbreviations: FD, frame displacement; HAMA, Hamilton Anxiety Rating Scale; and HAMD, Hamilton Depression Rating Scale. ^a^ represents ᵡ^2^ test, ^b^ for two sample *t*-tests, and ^c^ for Mann-Whitney *U* tests.

**Table S11.** Comparison of global network properties between patients with BSP and age-matched HCs (n = 102).

|  | **BSP (n=102; mean ± SD)** | **Age-matched HCs**  **(n=102; mean ± SD)** | ***T*** | **FDR corrected *P*** |
| --- | --- | --- | --- | --- |
| **a) BNA-274** | | | | |
| E_loc_ | 0.356 ± 0.005 | 0.358 ± 0.005 | -2.64 | 0.009^*^ |
| E_g_ | 0.268 ± 0.005 | 0.267 ± 0.005 | 1.98 | 0.048^*^ |
| C_p_ | 0.275 ± 0.012 | 0.280 ± 0.011 | -2.50 | 0.013^*^ |
| L_p_ | 0.793 ± 0.024 | 0.799 ± 0.023 | -1.44 | 0.151 |
| **b) AAL_116** | | | | |
| E_loc_ | 0.346 ± 0.007 | 0.347 ± 0.007 | -0.80 | 0.430 |
| E_g_ | 0.256 ± 0.008 | 0.252 ± 0.009 | 2.64 | 0.009^*^ |
| C_p_ | 0.277 ± 0.009 | 0.281 ± 0.010 | -2.56 | 0.011^*^ |
| L_p_ | 0.867 ± 0.047 | 0.886 ± 0.049 | -2.48 | 0.014^*^ |
| **c) rSchaefer-100** | | | | |
| E_loc_ | 0.348 ± 0.006 | 0.349 ± 0.006 | -0.63 | 0.522 |
| E_g_ | 0.260 ± 0.007 | 0.258 ± 0.007 | 1.81 | 0.072 |
| C_p_ | 0.274 ± 0.009 | 0.276 ± 0.010 | -1.74 | 0.082 |
| L_p_ | 0.840 ± 0.038 | 0.850 ± 0.038 | -1.52 | 0.131 |
| **d) rSchaefer-300** | | | | |
| E_loc_ | 0.356 ± 0.005 | 0.359 ± 0.005 | -2.82 | 0.005^*^ |
| E_g_ | 0.269 ± 0.005 | 0.268 ± 0.005 | 1.79 | 0.076 |
| C_p_ | 0.275 ± 0.012 | 0.280 ± 0.011 | -2.70 | 0.007^*^ |
| L_p_ | 0.788 ± 0.024 | 0.795 ± 0.024 | -1.52 | 0.130 |
| **e) rSchaefer-500** | | | | |
| E_loc_ | 0.358 ± 0.005 | 0.361 ± 0.005 | -3.37 | < 0.001 ^*^ |
| E_g_ | 0.272 ± 0.005 | 0.271 ± 0.004 | 1.95 | 0.052 |
| C_p_ | 0.274 ± 0.013 | 0.280 ± 0.012 | -2.94 | 0.004^*^ |
| L_p_ | 0.774 ± 0.020 | 0.779 ± 0.018 | -1.62 | 0.107 |
| **f) rSchaefer-700** | | | | |
| E_loc_ | 0.359 ± 0.006 | 0.362 ± 0.005 | -3.58 | < 0.001 ^*^ |
| E_g_ | 0.274 ± 0.004 | 0.272 ± 0.004 | 2.05 | 0.041^*^ |
| C_p_ | 0.274 ± 0.014 | 0.280 ± 0.013 | -3.10 | 0.002^*^ |
| L_p_ | 0.767 ± 0.018 | 0.772 ± 0.017 | -1.56 | 0.121 |

Abbreviations: BSP, blepharospasm; C_p_, clustering coefficient; E_g_, global efficiency; E_loc_, local efficiency; HCs, healthy controls; and L_p_, characteristic path length. **P* < 0.05.

**Table S12.** Comparison of global network properties between patients with BOD and age-matched HCs (n = 43).

|  | **BOD**  **(n=43; mean ± SD)** | **Age-matched HCs**  **(n=43; mean ± SD)** | ***T*** | **FDR corrected *P*** |
| --- | --- | --- | --- | --- |
| **a) BNA-274** | | | | |
| E_loc_ | 0.356 ± 0.006 | 0.358 ± 0.006 | -1.77 | 0.080 |
| E_g_ | 0.268 ± 0.005 | 0.266 ± 0.005 | 2.08 | 0.040^*^ |
| C_p_ | 0.274 ± 0.012 | 0.280 ± 0.013 | -1.99 | 0.050 |
| L_p_ | 0.792 ± 0.024 | 0.803 ± 0.022 | -1.94 | 0.055 |
| **b) AAL_116** | | | | |
| E_loc_ | 0.347 ± 0.010 | 0.347 ± 0.007 | -0.04 | 0.969 |
| E_g_ | 0.356 ± 0.006 | 0.252 ± 0.009 | 1.17 | 0.243 |
| C_p_ | 0.278 ± 0.010 | 0.280 ± 0.011 | -0.62 | 0.536 |
| L_p_ | 0.876 ± 0.052 | 0.887 ± 0.050 | -0.91 | 0.364 |
| **c) rSchaefer-100** | | | | |
| E_loc_ | 0.348 ± 0.008 | 0.349 ± 0.006 | -0.66 | 0.510 |
| E_g_ | 0.259 ± 0.007 | 0.258 ± 0.007 | 1.03 | 0.305 |
| C_p_ | 0.274 ± 0.009 | 0.276 ± 0.011 | -0.99 | 0.326 |
| L_p_ | 0.845 ± 0.040 | 0.852 ± 0.039 | -0.68 | 0.499 |
| **d) rSchaefer-300** | | | | |
| E_loc_ | 0.356 ± 0.006 | 0.358 ± 0.005 | -1.81 | 0.074 |
| E_g_ | 0.269 ± 0.004 | 0.267 ± 0.005 | 1.64 | 0.105 |
| C_p_ | 0.275 ± 0.011 | 0.279 ± 0.012 | -0.52 | 0.603 |
| L_p_ | 0.789 ± 0.022 | 0.797 ± 0.022 | -1.59 | 0.148 |
| **e) rSchaefer-500** | | | | |
| E_loc_ | 0.358 ± 0.006 | 0.360 ± 0.005 | -2.26 | 0.026^*^ |
| E_g_ | 0.272 ± 0.004 | 0.270 ± 0.004 | 2.11 | 0.038^*^ |
| C_p_ | 0.273 ± 0.130 | 0.279 ± 0.013 | -2.20 | 0.031^*^ |
| L_p_ | 0.773 ± 0.159 | 0.781 ± 0.017 | -2.03 | 0.046^*^ |
| **f) rSchaefer-700** | | | | |
| E_loc_ | 0.359 ± 0.007 | 0.362 ± 0.006 | -2.40 | 0.019^*^ |
| E_g_ | 0.273 ± 0.003 | 0.272 ± 0.004 | 1.90 | 0.061 |
| C_p_ | 0.273 ± 0.014 | 0.280 ± 0.013 | -2.29 | 0.025^*^ |
| L_p_ | 0.767 ± 0.014 | 0.773 ± 0.015 | -1.92 | 0.058 |

Abbreviations: BOD, blepharospasm-oromandibular dystonia; C_p_, clustering coefficient; E_g_, global efficiency; E_loc_, local efficiency; HCs, healthy controls; and L_p_, characteristic path length. **P* < 0.05.

**Table S13.** Comparison of global network properties between patients with CD and age-matched HCs (n = 56).

|  | **CD**  **(n=56; mean ± SD)** | **Aged-matched HCs**  **(n=56; mean ± SD)** | ***T*** | **FDR corrected *P*** |
| --- | --- | --- | --- | --- |
| **a) BNA-274** | | | | |
| E_loc_ | 0.357 ± 0.004 | 0.358 ± 0.005 | -1.05 | 0.298 |
| E_g_ | 0.269 ± 0.005 | 0.268 ± 0.004 | 0.16 | 0.877 |
| C_p_ | 0.276 ± 0.009 | 0.278 ± 0.012 | -0.60 | 0.547 |
| L_p_ | 0.789 ± 0.022 | 0.791 ± 0.019 | -0.048 | 0.962 |
| **b) AAL_116** | | | | |
| E_loc_ | 0.348 ± 0.006 | 0.348 ± 0.007 | -0.27 | 0.790 |
| E_g_ | 0.257 ± 0.008 | 0.255 ± 0.008 | 0.79 | 0.433 |
| C_p_ | 0.277 ± 0.009 | 0.279 ± 0.011 | -0.64 | 0.527 |
| L_p_ | 0.861 ± 0.043 | 0.870 ± 0.045 | -0.80 | 0.426 |
| **c) rSchaefer-100** | | | | |
| E_loc_ | 0.350 ± 0.005 | 0.349 ± 0.005 | 0.73 | 0.470 |
| E_g_ | 0.261 ± 0.006 | 0.260 ± 0.006 | 0.68 | 0.496 |
| C_p_ | 0.274 ± 0.009 | 0.274 ± 0.010 | 0.31 | 0.759 |
| L_p_ | 0.8316 ± 0.031 | 0.837 ± 0.032 | -0.49 | 0.628 |
| **d) rSchaefer-300** | | | | |
| E_loc_ | 0.358 ± 0.006 | 0.358 ± 0.005 | 0.23 | 0.820 |
| E_g_ | 0.270 ± 0.004 | 0.269 ± 0.004 | 0.38 | 0.707 |
| C_p_ | 0.276 ± 0.010 | 0.277 ± 0.011 | 0.02 | 0.988 |
| L_p_ | 0.785 ± 0.018 | 0.787 ± 0.020 | -0.39 | 0.694 |
| **e) rSchaefer-500** | | | | |
| E_loc_ | 0.359 ± 0.005 | 0.360 ± 0.005 | -0.47 | 0.638 |
| E_g_ | 0.273 ± 0.003 | 0.272 ± 0.004 | 0.47 | 0.642 |
| C_p_ | 0.275 ± 0.011 | 0.276 ± 0.013 | -0.27 | 0.790 |
| L_p_ | 0.771 ± 0.014 | 0.773 ± 0.015 | -3.63 | 0.717 |
| **f) rSchaefer-700** | | | | |
| E_loc_ | 0.360 ± 0.005 | 0.361 ± 0.006 | -0.55 | 0.587 |
| E_g_ | 0.274 ± 0.003 | 0.273 ± 0.003 | 0.53 | 0.600 |
| C_p_ | 0.276 ± 0.112 | 0.277 ± 0.013 | -0.48 | 0.630 |
| L_p_ | 0.766 ± 0.013 | 0.767 ± 0.014 | -0.33 | 0.743 |

Abbreviations: CD, cervical dystonia; C_p_, clustering coefficient; E_g_, global efficiency; E_loc_, local efficiency; HCs, healthy controls; and L_p_, characteristic path length.

**Table S14.** Differences in degree centrality and nodal efficiency based on BNA-274 atlas between patients with BSP and age-matched HCs (n = 102).

| **Labels** | **Region name** | **Abbreviations** | **BN** | **BSP (mean ± SD)** | **Age-matched** **HCs (mean ± SD)** | ***T*** | **FDR**  **correct *P*** |
| --- | --- | --- | --- | --- | --- | --- | --- |
| 1. **Degree centrality (BSP < HCs)** | | | | | | | |
| 163 | hypergranular insula | INS_L_6_1 | 2 | 34.831 ± 12.489 | 42.242 ± 12.369 | -3.98 | <0.05 |
| 1. **Degree centrality (BSP > HCs)** | | | | | | | |
| 231 | medial pre-frontal thalamus | Tha_L_8_1 | 8 | 37.687 ± 15.369 | 28.797 ± 15.903 | 3.74 | <0.05 |
| 237 | rostral temporal thalamus | Tha_L_8_4 | 8 | 30.231 ± 13.792 | 22.269 ± 13.821 | 3.84 | <0.05 |
| 238 |  | Tha_R_8_4 | 8 | 25.222 ± 14.440 | 17.818 ± 12.351 | 3.69 | <0.05 |
| 1. **Nodal efficiency (BSP < HCs)** | | | | | | | |
| 163 | hypergranular insula | INS_L_6_1 | 2 | 0.272 ± 0.029 | 0.288 ± 0.029 | -3.57 | <0.05 |
| 1. **Nodal efficiency (BSP > HCs)** | | | | | | | |
| 231 | medial pre-frontal thalamus | Tha_L_8_1 | 8 | 0.276 ± 0.041 | 0.252 ± 0.044 | 3.56 | <0.05 |
| 237 | rostral temporal thalamus | Tha_L_8_4 | 8 | 0.258 ± 0.039 | 0.234 ± 0.044 | 3.85 | <0.05 |
| 238 |  | Tha_R_8_4 | 8 | 0.245 ± 0.043 | 0.221 ± 0.043 | 3.72 | <0.05 |

Abbreviations: BNA-274, Brainnetome atlas with 274 brain regions; BSP, blepharospasm; and HCs, healthy controls.

**Table S15.** Comparison of global topological properties among the three dystonia subgroups (one-to-one and one-to-many) based on BNA-274 atlas, respectively.

|  | **BSP *v.s.* BOD** | | **BSP *v.s.* CD** | | **BOD *v.s.* CD** | | **BSP *v.s.* BOD &CD** | | **BOD *v.s.* BSP &CD** | | **CD *v.s.* BSP &BOD** | |
| --- | --- | --- | --- | --- | --- | --- | --- | --- | --- | --- | --- | --- |
|  | **T** | ***P*** | **T** | ***P*** | **T** | ***P*** | **T** | ***P*** | **T** | ***P*** | **T** | ***P*** |
| E_loc_ | -0.103 | 0.917 | 0.149 | 0.881 | -0.313 | 0.755 | 0.270 | 0.787 | 0.060 | 0.953 | -0.404 | 0.687 |
| E_g_ | 0.021 | 0.984 | -1.19 | 0.238 | -0.119 | 0.905 | -0.561 | 0.576 | -0.255 | 0.799 | 0.979 | 0.329 |
| C_p_ | 0.255 | 0.983 | 0.697 | 0.487 | -0.440 | 0.661 | 0.658 | 0.511 | -0.186 | 0.852 | -0.638 | 0.524 |
| L_p_ | 0.167 | 0.868 | 1.64 | 0.104 | 0.554 | 0.581 | 0.867 | 0.387 | 0.156 | 0.876 | -1.26 | 0.208 |

Abbreviations: BNA-274, Brainnetome atlas with 274 brain regions; BOD, blepharospasm-oromandibular dystonia; BSP, blepharospasm; CD, cervical dystonia; C_p_, clustering coefficient; E_g_, global efficiency; E_loc_, local efficiency; and L_p_, characteristic path length.

**Table S16.** Correlation between altered topological properties and motor severity and non-motor symptoms in the patients with BSP based on BNA-274 atlas.

| **Label** | **Region name** | **Abbreviations** | **JRS scores** | | **HAMA scores** | | **HAMD scores** | |
| --- | --- | --- | --- | --- | --- | --- | --- | --- |
|  |  |  | **R** | ***P*** | **R** | ***P*** | **R** | ***P*** |
| Global topological properties | | | | | | | | |
| E_loc_ | - | - | -0.111 | 0.272 | 0.021 | 0.838 | 0.118 | 0.272 |
| E_g_ | - | - | 0.197 | 0.051 | 0.023 | 0.820 | -0.029 | 0.790 |
| C_p_ | - | - | -0.156 | 0.123 | -0.019 | 0.851 | 0.075 | 0.486 |
| Degree Centrality (BSP < HCs) | | | | | | | | |
| 163 | hypergranular insula | INS_L_6_1 | -0.071 | 0.483 | -0.120 | 0.238 | -0.130 | 0.227 |
| Degree Centrality (BSP > HCs) | | | | | | | | |
| 231 | medial pre-frontal thalamus | Tha_L_8_1 | 0.086 | 0.395 | 0.059 | 0.559 | 0.024 | 0.822 |
| 232 |  | Tha_R_8_1 | 0.151 | 0.136 | 0.112 | 0.268 | 0.074 | 0.490 |
| 237 | rostral temporal thalamus | Tha_L_8_4 | 0.119 | 0.239 | 0.113 | 0.265 | 0.031 | 0.775 |
| 238 |  | Tha_R_8_4 | 0.133 | 0.189 | 0.117 | 0.250 | 0.052 | 0.663 |
| 245 | lateral pre-frontal thalamus | Tha_L_8_8 | 0.127 | 0.211 | -0.112 | 0.268 | -0.054 | 0.618 |
| 246 |  | Tha_R_8_8 | 0.115 | 0.256 | -0.049 | 0.631 | -0.025 | 0.818 |
| Nodal Efficiency (BSP > HCs) | | | | | | | | |
| 197 | ventromedial parietooccipital sulcus | MVOcC_L_5_5 | 0.203 | 0.044* | 0.117 | 0.248 | 0.106 | 0.328 |
| 227 | ventromedial putamen | BG_L_6_5 | 0.178 | 0.078 | 0.161 | 0.110 | 0.117 | 0.276 |
| 231 | medial pre-frontal thalamus | Tha_L_8_1 | 0.121 | 0.235 | 0.080 | 0.429 | 0.047 | 0.663 |
| 232 |  | Tha_R_8_1 | 0.190 | 0.060 | 0.124 | 0.223 | 0.091 | 0.399 |
| 237 | rostral temporal thalamus | Tha_L_8_4 | 0.177 | 0.080 | 0.127 | 0.212 | 0.049 | 0.648 |
| 238 |  | Tha_R_8_4 | 0.176 | 0.082 | 0.128 | 0.208 | 0.070 | 0.515 |
| 245 | lateral pre-frontal thalamus | Tha_L_8_8 | 0.159 | 0.117 | -0.081 | 0.428 | -0.028 | 0.795 |
| 246 |  | Tha_R_8_8 | 0.163 | 0.108 | -0.030 | 0.770 | 0.006 | 0.954 |
| 258 | - | Cerebellum_V_Crus_II | 0.070 | 0.494 | 0.107 | 0.291 | 0.104 | 0.335 |
| 270 | - | Cerebellum_V_IX | 0.239 | 0.017* | 0.016 | 0.871 | 0.026 | 0.806 |
| Nodal Clustering Coefficient (BSP < HCs) | | | | | | | | |
| 166 | ventral agranular insula | INS_LR_6_2 | -0.072 | 0.447 | -0.147 | 0.147 | -0.037 | 0.733 |
| 197 | ventromedial parietooccipital sulcus | MVOcC_L_5_5 | -0.308 | 0.002* | -0.007 | 0.941 | -0.002 | 0.985 |
| 198 |  | MVOcC_R_5_5 | -0.125 | 0.216 | 0.168 | 0.097 | 0.167 | 0.120 |
| 238 | rostral temporal thalamus | Tha_R_8_4 | 0.008 | 0.935 | -0.012 | 0.902 | 0.025 | 0.814 |
| 245 | lateral pre-frontal thalamus | Tha_L_8_8 | -0.072 | 0.479 | 0.016 | 0.878 | 0.031 | 0.774 |
| Nodal Local Efficiency (BSP < HCs) | | | | | | | | |
| 197 | ventromedial parietooccipital sulcus | MVOcC_L_5_5 | -0.014 | 0.893 | 0.048 | 0.635 | 0.040 | 0.710 |
| 238 | rostral temporal thalamus | Tha_R_8_4 | -0.052 | 0.612 | 0.025 | 0.807 | 0.146 | 0.750 |
| 245 | lateral pre-frontal thalamus | Tha_L_8_8 | -0.0068 | 0.506 | -0.084 | 0.410 | -0.084 | 0.438 |

Abbreviations: BNA-274, Brainnetome atlas with 274 brain regions; BSP, blepharospasm; C_p_, clustering coefficient; E_g_, global efficiency; E_loc_, local efficiency; HAMA, Hamilton Anxiety Rating Scale; HAMD, Hamilton Depression Rating Scale; HCs, healthy controls; JRS, Jankovic Rating Scale; and L_p_, characteristic path length. **P* < 0.05.

**Table S17.** Correlation between altered topological properties and motor severity and non-motor symptoms in the patients with BOD based on BNA-274 atlas.

| **Label** | **BFMDRS-M scores** | | **HAMA scores** | | **HAMD scores** | |
| --- | --- | --- | --- | --- | --- | --- |
|  | **R** | ***P*** | **R** | ***P*** | **R** | ***P*** |
| E_loc_ | -0.141 | 0.418 | 0.070 | 0.691 | -0.140 | 0.421 |
| E_g_ | 0.060 | 0.731 | 0.094 | 0.590 | 0.100 | 0.566 |
| C_p_ | -0.132 | 0.450 | 0.044 | 0.801 | -0.093 | 0.595 |
| L_p_ | -0.087 | 0.620 | -0.084 | 0.630 | -0.105 | 0.550 |

Abbreviations: BMFDRS-M, Burke-Fahn-Marsden Dystonia Rating Scale motor score; BNA-274, Brainnetome atlas with 274 brain regions; BOD, blepharospasm-oromandibular dystonia; C_p_, clustering coefficient; E_g_, global efficiency; E_loc_, local efficiency; HAMA, Hamilton Anxiety Rating Scale; HAMD, Hamilton Depression Rating Scale; HCs, healthy controls; and L_p_, characteristic path length.

**Table S18.** Correlation between altered topological properties and motor severity and non-motor symptoms in the patients with CD based on BNA-274 atlas.

| **Label** | **Region name** | **Abbreviations** | **TWSTRS scores** | | **HAMA scores** | | **HAMD scores** | |
| --- | --- | --- | --- | --- | --- | --- | --- | --- |
|  |  |  | **R** | ***P*** | **R** | ***P*** | **R** | ***P*** |
| Degree Centrality (CD > HCs) | | | | | | | | |
| 4 | dorsolateral area 8 | SFG_R_7_2 | 0.175 | 0.245 | 0.166 | 0.234 | 0.267 | 0.055 |
| 231 | medial pre-frontal thalamus | Tha_L_8_1 | 0.006 | 0.969 | 0.234 | 0.091 | 0.251 | 0.072 |
| 232 |  | Tha_R_8_1 | 0.088 | 0.559 | 0.235 | 0.090 | 0.258 | 0.065 |
| 233 | pre-motor thalamus | Tha_L_8_2 | 0.083 | 0.584 | 0.132 | 0.345 | 0.059 | 0.678 |
| 246 | lateral pre-frontal thalamus | Tha_R_8_8 | 0.085 | 0.576 | 0.131 | 0.350 | 0.165 | 0.244 |
| 268 |  | Cerebellum_R_VIIIb | -0.081 | 0.593 | 0.011 | 0.936 | 0.005 | 0.972 |
| 270 |  | Cerebellum_V_IX | -0.055 | 0.717 | -0.134 | 0.339 | -0.160 | 0.256 |

Abbreviations: BNA-274, Brainnetome atlas with 274 brain regions; CD, cervical dystonia; HAMA, Hamilton Anxiety Rating Scale; HAMD, Hamilton Depression Rating Scale; HCs, healthy controls; and TWSTRS; Toronto Western Spasmodic Torticollis Rating Scale.
